# An improved transcriptome annotation reveals asymmetric expression and distinct regulation patterns in allotetraploid common carp

**DOI:** 10.1038/s42003-024-07177-3

**Published:** 2024-11-20

**Authors:** Qi Wang, Meidi Huang Yang, Shuangting Yu, Yingjie Chen, Kaikuo Wang, Yan Zhang, Ran Zhao, Jiongtang Li

**Affiliations:** Key Laboratory of Aquatic Genomics, Ministry of Agriculture and Rural Affairs, and Beijing Key Laboratory of Fishery Biotechnology, Chinese Academy of Fishery Sciences, Beijing, China

**Keywords:** Genome, Polyploidy

## Abstract

In allotetraploid common carp, protein-coding homoeologs presented divergent expression levels between the two subgenomes. However, whether subgenome dominance occurs in other transcriptional and post-transcriptional events remains unknown. Using Illumina RNA sequencing and PacBio full-length sequencing, we refined the common carp transcriptome annotation and explored differences in four transcriptional and post-transcriptional events between the two subgenomes. The results revealed that the B subgenome presented more alternative splicing events, as did lncRNAs and circRNAs. However, the expression levels, tissue specificity, sequence features, and functions of lncRNAs and circRNAs did not significantly differ between the two subgenomes, suggesting a common regulatory mechanism shared by the two subgenomes. Furthermore, both the number and base substitution frequency of RNA editing events were greater in the B subgenome. Functional analyses of these transcriptional events also revealed subgenome bias. Genes that undergo alternative splicing in the A subgenome participate in more biological processes, and lncRNA targets show a preference between subgenomes. CircRNA host genes in the B subgenome were associated with more biological functions, and RNA editing preferentially occurred in noncoding regions or led to nonsynonymous mutations in the B subgenome. Taken together, the refined transcriptome annotation revealed complicated and imbalanced expression strategies in allotetraploid common carp.

## Abstract (web summary)

Comprehensive transcriptomic analysis revealed distinct patterns in expression, regulation, and function of alternative splicing, lncRNA, circRNA,and RNA editing events between the two subgenomes of allotetraploid common carp.

## Introduction

Polyploidization plays a significant role in driving species formation and enhancing environmental adaptability. However, allopolyploids formed through interspecific hybridization often exhibit incompatibility between genetic material derived from different parental sources. Sequence variation, functional diversification, and differential expression levels of duplicated genes are involved in their regulation^1,2^. In numerous polyploids, genes from different subgenomes exhibit divergent expression levels^3–6^. One of the subgenomes commonly exhibited markedly stronger expression than the other subgenomes did, a phenomenon known as subgenome dominance. The dominant subgenome typically exhibits fewer gene losses, chromosome fusion/fission events, and stronger purifying selection^7^. The presence of these asymmetrical gene expression patterns is crucial for mitigating the detrimental effects of subgenome incompatibility and enhancing the adaptability of hybrid species^2,8,9^. In addition to protein-coding genes, subgenome dominance is also observed in small RNA targeting^10^, RNA modification^11^, and protein translation efficiency^12,13^. These differential transcriptional and post-transcriptional modifications across subgenomes jointly regulate the biological function of duplicated genes, thereby maintaining genome stability.

Whole-genome duplication (WGD) events are rare in animals. Several independent WGD events have been identified in cyprinids^14^. Cyprinids are also the most widely farmed fish worldwide. The common carp (*Cyprinus carpio*), a model species of *Cyprinidae*, underwent a fourth-round allotetraploidization event 14.4 million years ago^15^. The common carp genome consists of 25 pairs of homologous chromosomes, designated A1-A25 and B1-B25 based on their subgenome affiliations. Unlike those in other polyploids, the two subgenomes in common carp exhibit parallel structures, characterized by equivalent chromosome components, similar transposon contents, and symmetric purifying selection^4^. However, subgenome dominance is still observed in the expression of protein-coding genes in common carp^4,16^. Several other polyploids within *Cyprinidae* also show divergent expression across subgenomes. Notably, a clear bias in tRNA and rRNA gene frequency between subgenomes was observed in Prussian carp^17^. In addition, studies on interspecific hybridization within *Cyprinidae* have shown that subgenome dominance is established rapidly in the early stages of hybridization and then strengthened over time^6,18^. This asymmetric expression contributes to embryo development^6^ and dietary adaptation^19^. However, these studies have typically focused only on protein-coding genes. It remains uncertain whether subgenome dominance occurred during other transcriptional and post-transcriptional processes in common carp.

Long noncoding RNAs (lncRNAs) play active roles in a variety of biological processes, including dosage compensation, genomic imprinting, and genomic stability, by interacting with DNA, RNA, and proteins^20^. Compared with protein-coding genes, lncRNAs have low sequence conservation and expression levels with high tissue specificity^21^. Circular RNA (circRNA) is another type of noncoding RNA. Through binding to miRNAs and proteins, circRNAs are involved in gene expression regulation and participate in the immune response, growth, metabolism, and development in teleosts^22,23^. RNA editing modifies nucleotides in RNAs through base substitution, forming new transcripts whose sequence differs from that of the DNA template. This process participates in regulating protein-coding gene function and stability^24^. However, the expression divergence of these three transcriptional events across subgenomes in polyploids remains unclear.

To answer these questions, we report an updated common carp transcriptome annotation via PacBio full-length transcriptome sequencing and Illumina RNA sequencing, and generate a comprehensive expression landscape, including alternative splicing (AS), lncRNA, circRNA, and RNA editing in nine organs. We compared the abundance of these transcriptional and post-transcriptional events between the two subgenomes and investigated their functional differences. These distinctive transcriptional and post-transcriptional expression patterns provide valuable insights into the complex expression divergence strategies and subgenome evolution in the allotetraploid common carp.

## Results

### Improved transcriptome annotation of common carp

We obtained 1,808,386 subreads (4.73 Gb) with an N50 length of 3,094 bp by SMRT sequencing (Fig. S1A). After error correction and redundancy removal, we obtained 201,787 circular consensus sequence (CCS) reads with an average length of 3110 bp (Table S1). The average depth of CCS reads was 5.51 passes, and the average quality score was 24.12. After polishing with Illumina RNA-seq reads, 91.38% of all long reads were successfully aligned to the common carp reference genome with a median coverage of 95.57%.

By integrating the PacBio full-length transcriptome sequencing data with the Illumina RNA-seq data from nine organs, we generated a high-confidence dataset comprising 61,505 genes and 140,233 alternative splicing transcripts (Fig. S2). Among these, 130,348 isoforms from 55,503 genes had protein-coding potential (Fig. S2**)**. We implemented functional annotation of these protein-coding isoforms based on the homology-based alignment. There were 98,916 (75.89%) and 100,904 isoforms (77.41%) annotated by the KOBAS server and Swiss-Prot database, respectively (Table S2). After merging functional annotations, 110,497 (84.77%) isoforms from 43,905 (79.10%) protein-coding genes were annotated with potential biological functions.

The B subgenome contained 29,453 genes, which was more than 27,723 genes in the A subgenome. However, this difference was not statistically significant (*χ*^2^ test, *P* = 0.29), likely due to the variation in chromosome length between the two subgenomes. The protein-coding genes were also evenly distributed across both subgenomes (A subgenome: 25,285; B subgenome: 26,583; *χ*^2^ test, *P* = 0.85). Additionally, 4329 genes and 8306 transcripts were located on scaffolds that were not anchored on chromosomes, of which 7112 transcripts derived from 3635 genes had protein-coding potential.

**Fig. 1:**
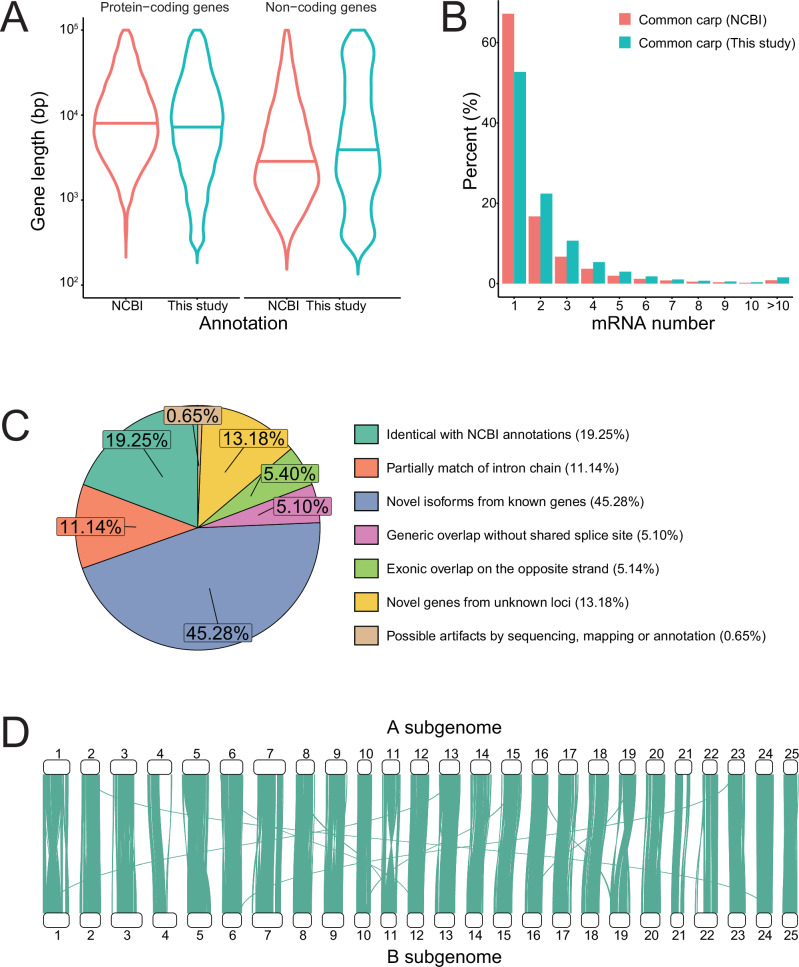
A high-quality transcriptome annotation of common carp by integrating PacBio sequencing and Illumina RNA-seq data. **A** The length distribution of protein-coding genes was similar between the two annotations (Wilcoxon rank-sum test, *P* = 0.12). However, the noncoding genes in the updated annotation were significantly longer (Wilcoxon rank-sum test, *P* < 2.20 × 10^−16^). **B** The percentage of genes transcribed multiple isoforms in the updated annotation was significantly greater than that in the NCBI reference annotation (*χ*^2^ test, *P* < 2.20 × 10^−16^). **C** Isoforms in the updated annotation were classified into seven groups, as compared with the NCBI reference annotation of common carp. **D** High collinearity was observed between the two subgenomes of common carp.

We compared the new annotation against the NCBI reference annotation to assess the quality of the updated annotation. First, the gene completeness of the transcriptome annotation was evaluated with BUSCO. The updated annotation had a lower missing rate (0.7%) and a higher completeness rate (98.8%) than the NCBI reference annotation (2.0% and 97.2%) and previous annotation^4^ (6.51% and 91.90%, Table S3), suggesting an increase in annotation quality. Second, we aligned coding sequences to annotated proteins in the closely related species *Paracanthobrama guichenoti*, *Puntius tetrazona*, and *Danio rerio* (Table S4). Over 85% of protein-coding genes in common carp were found to have homoeologs in related species, highlighting the reliability of the updated annotations. Furthermore, we compared the gene structures between the updated annotation and the NCBI reference annotation. The lengths of protein-coding genes were equivalent in both annotations, whereas the noncoding genes in the updated annotation were much longer than those in the NCBI annotation (Fig. 1A, Wilcoxon rank-sum test, *P* < 2.20 × 10^−16^). Although the CDS lengths in the updated annotation (median: 1125 bp) were shorter than those in the NCBI annotation (median: 1491 bp, Wilcoxon rank-sum test, *P* < 2.20 × 10^−16^), they were still comparable to those reported in the model species zebrafish (NCBI accession: GCA_000002035.4, median: 1095 bp, Wilcoxon rank-sum test, *P* = 0.93, Fig. S3A). Both annotations exhibited similar distributions of exon and intron lengths (Fig. S3B, C). Interestingly, the number of isoforms per gene was significantly greater in the updated annotation (Fig. 1B and Table S5, *χ*^2^ test, *P* < 2.20 × 10^−16^). To better evaluate the annotation quality, we classified all the isoforms into seven groups based on the match of gene structure to the NCBI reference annotation (Fig. 1C). Only 910 isoforms were marked as possible artifacts due to the intron match on different strands, pre-mRNA fragments and polymerase run-on. Almost one-third of the isoforms were either completely identical or partially matched the intron chain to the NCBI annotation. Novel isoforms from known genes accounted for more than 45% of all isoforms, and 18,482 (13.18%) novel isoforms were derived from unknown loci that were absent in the NCBI annotation. We further compared the sequence and expression features of each group. Excluding possible artifacts, more than 80% of the isoforms in the other groups consisted of multiple exons, and the classical “GT-AG” splicing signal was detected at more than 95% of intron sites (Fig. S4). There were also no significant differences in expression levels among these annotated groups (Fig. S5A, Wilcoxon rank-sum test, *P* > 0.05), and similar tissue-specific expression patterns were observed (Fig. S5B). In addition, comparisons of sequence identity with zebrafish proteins across these groups (Fig. S6A), as well as expression analyses of other reported RNA-seq data (Fig. S6B), confirmed the authenticity of these novel isoforms and genes. Overall, these data highlighted the high-quality of the updated transcriptome annotation.

Furthermore, high collinearity between the two subgenomes of common carp was still observed based on the updated annotation (Fig. 1D). The A and B subgenomes shared 17,710 syntenic gene pairs (60.13% for the A subgenome, 63.88% for the B subgenome), which was 1827 more pairs than previously reported^4^.

### More alternative splicing events in the B subgenome with higher tissue specificity

**Fig. 2:**
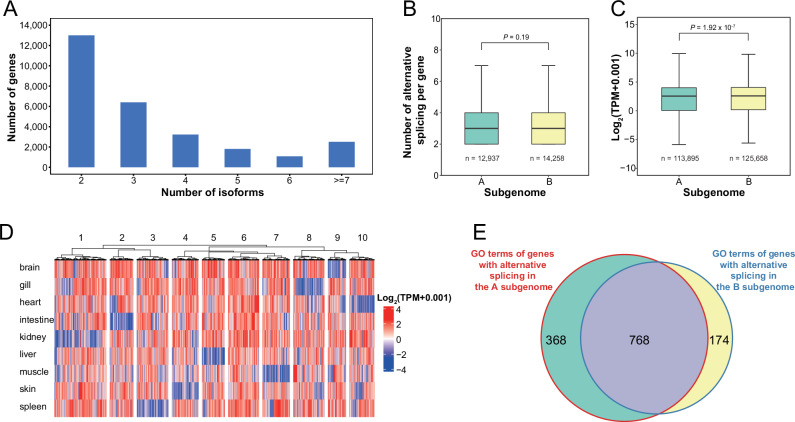
Summary of AS in the two subgenomes and nine organs. **A** Histogram showing the distribution of isoform counts per gene. **B** There was no significant difference in the number of isoforms per gene between the A and B subgenomes (Wilcoxon rank-sum test). **C** Compared with those in the A subgenome, the expression levels of genes with AS were significantly higher in the B subgenome (Wilcoxon rank-sum test). **D** The expression heatmap of 6024 isoforms shared across nine organs illustrates the tissue-specificity of AS. **E** Venn diagram showing the intersection of GO terms enriched in protein-coding genes underwent AS in the A and B subgenomes.

Among all protein-coding genes, 28,460 (51.28%) underwent alternative splicing (AS). Approximately half of these genes produced only two isoforms, whereas fewer than one in ten genes yielded more than seven isoforms (Fig. 2A). In total, AS in common carp produced 106,016 isoforms with an average of 3.73 isoforms per gene, which was higher than that observed in other teleosts (averages of 3.28 and 3.64 isoforms per gene in zebrafish^25^ and rainbow trout^26^, respectively).

The average frequency of AS per gene was similar in both subgenomes (Wilcoxon rank-sum test, *P* = 0.1895; Fig. 2B). In contrast, the number and proportion of protein-coding genes with AS in the B subgenome (14,258 and 53.64%) were significantly greater than those in the A subgenome (12,937 and 51.16%, *χ*^2^ test, *P* = 1.87 × 10^−8^). Specifically, there were significantly more protein-coding genes with AS in B7, B6, B5, and B22 than in their homoeologous counterparts in the A subgenome (*χ*^2^ test, *P* = 3.13 × 10^−16^ ~ 5.31 × 10^−3^; Fig. S7). This pattern was consistent across all nine examined organs, where the number and proportion of protein-coding genes with AS were always greater in the B subgenome than in the A subgenome (Table S6). To account for the differences in gene number, we compared homoeologous gene pairs and found that a greater percentage of homoeologs in the B subgenome (65.74%) underwent AS than their counterparts in the A subgenome (62.69%, *χ*^2^ test, *P* = 2.31 × 10^−9^). In nearly half of these cases (8678, 49.00%), AS occurred simultaneously in homoeologs from both subgenomes (Fig. S8). Additionally, 2425 (13.69%) and 2965 (16.74%) homoeologs underwent subgenome-specific AS in the A and B subgenomes, respectively. In summary, these data suggested that a greater proportion of protein-coding genes underwent AS in the B subgenome, whereas the average AS frequency was comparable between the two subgenomes.

By analyzing the relationship between AS and gene abundance, we found that protein-coding genes generating more isoforms tended to have higher expression levels (Fig. S8). In addition, the expression levels of the protein-coding genes with AS in the B subgenome (median Transcripts Per Million = 6.02) were significantly higher than the A subgenome (median Transcripts Per Million = 5.82; Wilcoxon rank-sum test, *P* = 1.92 × 10^−7^; Fig. 2C). This trend was confirmed in genes that produced different numbers of isoforms (Fig. S8).

We also investigated the expression profiles of AS events in common carp. The most (28,507, 27.78%) isoforms were expressed simultaneously in seven organs, followed by 19,650 (19.15%) and 6024 (5.87%) isoforms in eight and nine organs, respectively (Fig. S10A). Among the 1352 tissue-specific isoforms that were expressed in only one organ, the vast majority (1067, 78.92%) were expressed only in the brain (Fig. S9B). Additionally, we examined the expression levels of isoforms shared by nine organs. The 6024 isoforms were clustered into ten groups (Fig. 2D). Except for the protein-coding genes in the sixth group, which were expressed at high levels in all organs, the other nine groups of genes were low-expressed in only one of the examined organs.

AS is an important driver of increased protein diversity. Based on differences in coding sequence from multiple isoforms originating from the same gene, we classified 28,460 genes with AS into three groups (Table S7). (1) Protein sequences were not affected by AS. There were 2748 genes belonging to this group. These genes were enriched in 15 Gene Ontology (GO) terms, most of which were associated with ribosome and translational processes (Table S9). (2) All isoforms of the same gene encode different protein sequences. Most protein-coding genes with AS (67.57%) belong to this group. (3) Parts of isoforms encoded different protein sequences, comprising 22.78% of all protein-coding genes with AS. We compared the gene distribution in these groups and found no significant difference in the effect of AS on protein diversity across the two subgenomes (Table S7, *χ*^2^ test, *P* = 0.72).

We further compared the functions of genes that underwent AS in the A and B subgenomes. The protein-coding genes with AS in the A subgenome were enriched in 1136 GO terms, which was significantly greater than those in the B subgenome (942, *χ*^2^ test, *P* = 5.38 × 10^−10^). There were 768 GO terms shared by both, which was significantly higher than expected from two independent random samples (Fig. 2E**;** hypergeometric test, *P* < 2.2 × 10^−16^). These common GO terms were related to development, metabolism, and regulation. Another 368 GO terms that were specifically enriched in the A subgenome were associated mainly with substance exchange and signal transport. In contrast, 174 GO terms that were specifically enriched in the B subgenome were primarily involved in the activation of immune system and response to stimuli (Fig. S11).

We also compared the ability of the two sequencing technologies to detect AS events. The numbers of AS events identified by PacBio sequencing and Illumina sequencing were 28,802 and 25,248, respectively. Both methods shared most of the AS events (52.98% and 60.44% for AS events detected by PacBio and Illumina sequencing, respectively, Fig. S12A). The number of AS events specifically detected by PacBio sequencing was 13,542, which was significantly greater than that detected only by Illumina sequencing (9988, *χ*^2^ test, *P* < 2.20 × 10^−16^). On the other hand, the AS events detected by each method showed different positional distribution patterns. The proportion of AS events identified by PacBio sequencing in the CDS region was significantly lower than that identified by Illumina sequencing (40.75% vs. 63.57%, *χ*^2^ test, *P* < 2.20 × 10^−16^; Fig. S12B). In contrast, the proportions of AS events in the 5’ UTR and 3’ UTR detected by PacBio sequencing (31.49% and 27.76%, respectively) were significantly greater than those detected by Illumina sequencing (22.69% and 13.74%, *χ*^2^ test, *P* < 2.20 × 10^−16^).

### More lncRNAs in the B subgenome, and biased functions between the two subgenomes

Among 9885 transcripts without predicted open reading frames, 8012 transcripts from 6000 genes were identified as lncRNAs. Our annotation identified 328 additional lncRNAs compared with the NCBI reference annotation, which included 7684 lncRNAs. Almost two-thirds of the lncRNAs (5165, 64.47%) were longer than 500 bp (Fig. S13). Relatively few lncRNAs (1135, 18.9%) were transcribed from protein-coding genes, whereas the majority (88.23%) originated from synteny blocks (Fig. S14).

**Fig. 3:**
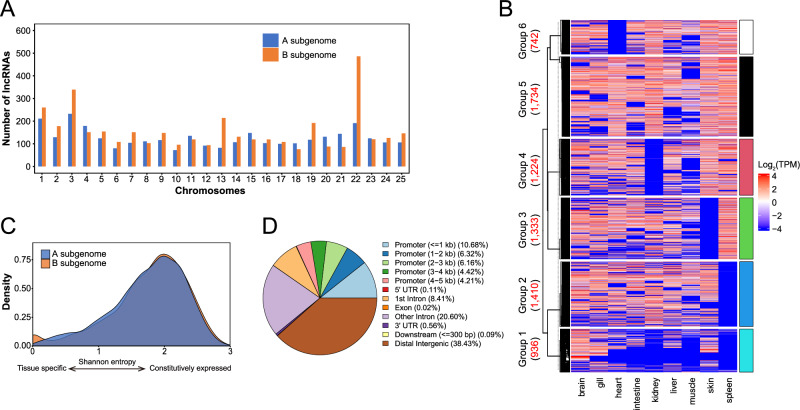
Expression and functions of lncRNAs in common carp. **A** The B subgenome harbored a greater number of lncRNAs than the A subgenome. **B** Heatmap of expression level showing the tissue-specificity of lncRNAs. The numbers within parentheses indicate the number of lncRNAs in each group. **C** LncRNAs originating from the A and B subgenomes showed similar tissue specificity. Shannon entropy equals log_2_(number of organs) when the gene expressed uniformly in all organs, and equals zero if gene expressed in a single organ. **D** Distribution of potential lncRNA target sites across genic elements.

We analyzed the chromosome distributions of these lncRNAs. A total of 953 lncRNAs, corresponding to 635 genes, were in unanchored scaffolds (Fig. S2). More lncRNAs were identified in the B subgenome (3912) than in the A subgenome (3147). In more detail, 17 out of 25 chromosomes (68.00%) in the B subgenome harbored more lncRNAs than their counterparts in the A subgenome (Fig. 3A).

The majority (89.00%) of the lncRNAs were expressed in at least three organs and 124 (1.55%) lncRNAs were expressed in only one organ. It is noteworthy that the expression levels of the lncRNAs were comparable to those of mRNAs in common carp (Fig. S15A). Based on their abundance, all lncRNAs were classified into six groups (Fig. 3B), which presented relatively constant expression levels across nine organs. Furthermore, as estimated using the Shannon entropy, the lncRNAs were more likely to be constitutively expressed in all organs, and the tissue-specificity of lncRNAs was equivalent to that of mRNAs (Fig. S15B).

We also compared the expression profiles of lncRNAs in the two subgenomes. There was no significant difference in the abundance of lncRNAs between the A and B subgenomes in any of the examined organs (Wilcoxon rank-sum test, *P* > 0.05; Fig. S16). Additionally, the tissue-specificity of lncRNAs was almost equivalent between the two subgenomes (Fig. 3C).

We further compared the putative functions of lncRNAs from the two subgenomes using multiple methods. First, we performed Gene Ontology (GO) analysis on the genes that produced lncRNAs and protein-coding genes simultaneously. Compared with those in the A subgenome (39), the lncRNA host genes in the B subgenome presented more enriched GO terms (482). Surprisingly, the lncRNA host genes in the two subgenomes shared 30 enriched GO terms (Fig. S17A). These common GO terms were mainly related to immune responses, including “response to biotic stimulus (GO:0009607)”, “immune effector process (GO:0002252)”, and “B cell-mediated immunity (GO:0019724)”. The enriched GO terms specific to the A subgenome were also related to immune responses, including “granulocyte migration (GO:0097530)”, “negative regulation of T cell proliferation (GO:0042130)” and “negative regulation of leukocyte proliferation (GO:0070664)” (Fig. S17B). In contrast, lncRNA host genes in the B subgenome primarily contribute to signaling and other immune responses, such as “I-kappaB kinase/NF-kappaB signaling (GO:0007249)”, “MHC class I biosynthetic process (GO:0045341)”, “Toll signaling pathway (GO:0008063)”, “angiogenesis (GO:0001525)” and “locomotion (GO:0040011)” (Fig. S17C).

Potential genomic and mRNA targets of the lncRNAs were predicted to further explore the functional differences of the lncRNAs between the two subgenomes. Among the 11,847,546 predicted potential lncRNA‒genomic interactions, the majority of the targets were located in distal intergenic regions (38.43%), promoters (31.79%), and introns (29.01%, Fig. 3D). Fewer interactions were observed in the UTRs (0.66%), downstream (0.09%) and CDS regions (0.02%). By comparing the genomic distribution of these targets, we found that the lncRNAs from the A subgenome had a greater number of potential genomic target sites (61.88%) compared to the lncRNAs from the B subgenome (34.35%, Table S9), despite there being fewer lncRNAs in the A subgenome. The A subgenome also exhibited a higher proportion of lncRNA targets in intergenic regions and UTRs than the B subgenome, regardless of the lncRNA’s origin. Conversely, lncRNA‒genomic interactions targeted intron showed greater prevalence in the B subgenome. LncRNA-mRNA interaction pairs were also predicted. The majority of lncRNA‒mRNA pairs (90.63%) presented divergent expression patterns with large Euclidean distances and low correlations, whereas 8.69% of lncRNA‒mRNA pairs were detected with highly correlated expression levels (Fig. S18). The lncRNAs in the B subgenome preferentially targeted more mRNAs (median: 16) compared to the lncRNAs in the A subgenome (median: 13, Wilcoxon rank-sum test, *P* = 0.0098). Furthermore, the intra-subgenome lncRNA-mRNA pairs were more prevalent in the B subgenome (54.91%) than in the A subgenome (48.09%, *χ*^2^ test, *P* < 2.20 × 10^−16^, Fig. S19**)**.

### More circRNAs in the B subgenome with equivalent expression levels between the two subgenomes

**Fig. 4:**
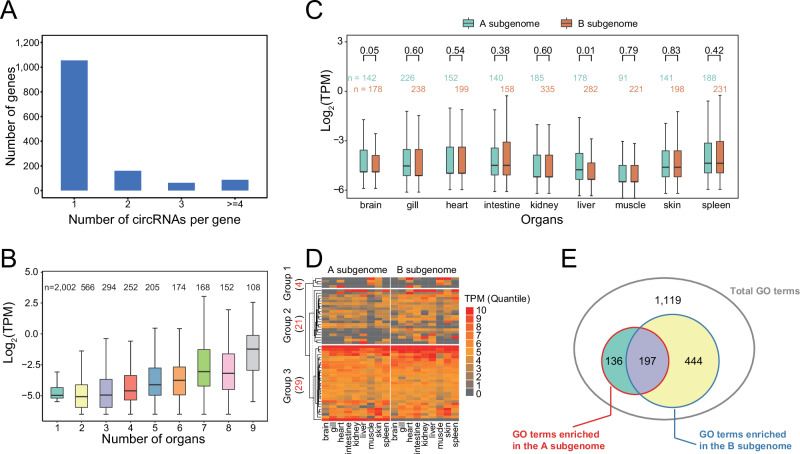
Balance of circRNA expression levels and functions between two subgenomes in common carp. **A** Histogram showing the distribution of host genes that produced varying numbers of circRNAs. **B** CircRNA abundance was positively correlated with the number of organs in which circRNAs were expressed (Spearman rank correlation analysis, *P* < 2.20 × 10^−16^, *r* = 0.29). **C** There was no significant difference in the expression levels of circRNAs between the A and B subgenomes (Wilcoxon rank-sum test). **D** Homoeologous genes that can produce circRNAs were expressed at approximately equal levels. The number within parentheses represents the number of homoeologous genes with circRNAs. **E** Venn diagram showing the intersection of GO terms enriched in circRNA host genes in the A and B subgenomes.

We identified 2571 circRNAs derived from 1365 host genes. Most of the host genes (1055, 77.29%) produced only one circRNA, whereas 310 host genes (22.71%) generated multiple circRNAs through alternative back-splicing (Fig. 4A). A total of 1797 (69.89%) circRNAs originated from introns, and 774 (30.11%) originated from exons.

There were 204 circRNAs transcribed from 58 host genes, located in scaffolds that were not anchored on chromosomes. The number of circRNAs from the A subgenome (948 corresponding to 605 host genes, accounting for 36.87%) was significantly lower than that from the B subgenome (1419 corresponding to 702 host genes, 55.19%, *χ*^2^ test, *P* < 2.20 × 10^−16^; Fig. S2). The primary contributors to these differences were the intronic circRNAs, which numbered 593 and 1,092 in the A and B subgenomes (*χ*^2^ test, *P* = 2.18 × 10^−7^), respectively. A closer examination of expressed circRNAs from two subgenomes in nine organs confirmed that circRNAs were more prevalent in the B subgenome (Fig. S20). Reverse complementary matches and transposons have been proposed as important factors promoting circRNA biogenesis^27^. To explain the observed preference for the B subgenome in circRNA formation, we evaluated the distribution of these functional elements across the two subgenomes. Surprisingly, circRNAs derived from the A and B subgenomes exhibited similar flanking intron lengths, reverse complementary matches, and transposon distributions (Wilcoxon rank-sum test, *P* > 0.01; Figs. S21 and S22).

CircRNAs exhibited tissue-specific expression patterns. The majority of circRNAs (2002, and 77.87%) were expressed in only a single organ, and only 12 circRNAs were detected across all nine organs (Fig. S23A, B). Furthermore, the expression levels of circRNAs were positively correlated with the number of organs in where circRNAs were expressed (Spearman rank correlation analysis, *P* < 2.20 × 10^−16^, *r* = 0.29). CircRNAs detected in more organs presented higher expression levels (Fig. 4B).

We also compared the expression patterns of circRNAs and host genes between the two subgenomes. No significant difference was found in the abundance of circRNAs between the two subgenomes (Fig. 4C, Wilcoxon rank-sum test, *P* > 0.05). Although the overall gene expression levels in the B subgenome were higher, the abundances of circRNA host genes were equivalent between the two subgenomes (Wilcoxon rank-sum test, *P* = 0.27). When examining homoeologs that produced circRNAs in the A and B subgenomes, we also found that their expression levels were largely comparable (Fig. 4D). Additionally, the expression levels of circRNAs and their corresponding host genes were not correlated in the two subgenomes, except for a weak positive correlation in circRNAs from the A subgenome in the spleen (Table S10, Spearman rank correlation analysis, *P* = 0.003, *r* = 0.22). Collectively, these results suggest that gene abundance does appear to be a major contributor to the observed differences in circRNA expression between the two subgenomes.

To further explore the functional differences, we compared the GO annotations of circRNA host genes between the two subgenomes. In the A subgenome, circRNA host genes were enriched in 333 GO terms, mostly related to metabolism and development (Table S11). The circRNA host genes located in the B subgenome, in contrast, were associated with more biological functions, with a total of 641 GO terms related to signal transduction and regulation (Table S12). Interestingly, the two subgenomes shared 197 GO terms (Fig. 4E), and the majority of which were involved in immune and stress responses. Additionally, GO terms such as “RNA binding (GO:0003723)”, “positive regulation of RNA splicing (GO:0033120)”, “spliceosomal complex (GO:0005681)” and “mRNA metabolic process (GO:0016071)” were common to both subgenomes, suggesting that genes involved in RNA transcription and splicing processes are more likely to produce circRNAs.

### Greater number of RNA editing sites and higher frequency of base substitutions in the B subgenome

A total of 194,263 RNA editing sites were detected across nine organs. These sites were involved in 24,523 genes, including 23,313 protein-coding genes and 1210 lncRNA genes. Twelve types of base substitutions were identified. The two most frequent types of RNA editing were canonical A-to-G (40,826, 21.02%) and C-to-T conversion (28,579, 14.71%, Fig. S24A). However, the number of genes undergoing non-canonical G-to-A conversion was the highest, at 13,303. This was followed by 13,069 genes exhibiting canonical C-to-T conversion and 11,682 genes undergoing canonical A-to-G conversion (Fig. S24B). Furthermore, the base substitution frequencies among these canonical and non-canonical conversions were almost equivalent (Fig. S24C).

RNA editing exhibited strong tissue-specific patterns. Over half of the sites (54.16%) were detected in only one organ, while a small fraction (1173, 0.60%) was shared across all nine organs **(**Fig. S25). The brain exhibited the highest RNA editing activity with 74,381 sites and the highest percentage of organ-specific RNA editing sites (42.16%). In contrast, muscle had the fewest organ-specific sites (1960, Table S13).

**Fig. 5:**
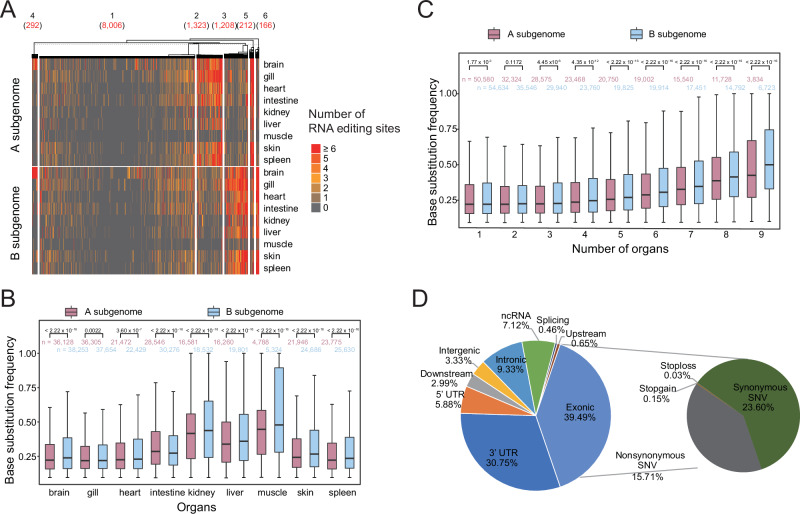
Characterization of RNA editing in common carp. **A** Heatmap of the number of RNA editing sites occurring on homoeologous genes. The number within parentheses represents the number of homoeologous gene pairs. **B** The base substitution frequency of RNA editing was higher in the B subgenome compared to the A subgenome (Wilcoxon rank-sum test). **C** The base substitution frequency of RNA editing was positively correlated with the number of organs in which RNA editing sites were detected (Spearman rank correlation analysis, *P* < 2.20 × 10^−16^, *r* = 0^.^20 fo*r* the A subgenome, *P* < 2.20 × 10^−16^, *r* = 0.25 for the B subgenome). **D** Distribution of genic annotation for RNA editing sites.

More RNA editing sites were observed in the B subgenome (100,700) than in the A subgenome (93,563). Examination across all nine organs confirmed that RNA editing was preferred in the B subgenome (Fig. S26A; *χ*^2^ test, *P* < 2.20 × 10^−16^). Furthermore, a greater number of genes (12,637) in the B subgenome than in the A subgenome (11,886) underwent RNA editing. However, the number of RNA editing sites per gene was equivalent between the two subgenomes (Fig. S26B, Wilcoxon rank-sum test, *P* = 0.21). We further compared RNA editing activity in homoeologous gene pairs undergoing RNA editing. All homoeologs were divided into six clusters according to the number of RNA editing sites (Fig. 5A). The first and fourth clusters exhibited equivalent numbers of RNA editing sites between the two subgenomes, accounting for 71.44% and 2.61% of the homoeologs, respectively. In the second and fifth clusters, 13.70% of the homoeologs had more RNA editing sites in the A subgenome than their counterparts. Conversely, 13.29% of the homoeologs in the third and fifth clusters had more RNA editing sites in the B subgenome than in the A subgenome.

We compared the base substitution frequency of RNA editing sites between the two subgenomes. Across all organs except for intestine, the base substitution frequency was higher than in the B subgenome compared with the A subgenome (Wilcoxon rank-sum test, *P* < 0.01; Fig. 5B). Additionally, the base substitution frequency was positively correlated with the number of detected organs where RNA editing was detected in the A subgenome (*P* = 7.50 × 10^−4^, *r* = 0.933) and the B subgenome (*P* = 3.50 × 10^−4^, *r* = 0.950; Fig. 5C). The base substitution frequency at RNA editing sites was consistently higher in the B subgenome compared to the A subgenome, except for sites shared by only two organs (Wilcoxon rank-sum test, *P* values listed in Fig. 5C). A closer examination of homoeologs also revealed a significantly higher substitution frequency in the B subgenome than in their counterparts (*P* values listed in Fig. S27). This trend was also observed for the 1173 RNA editing sites shared across nine organs (Fig. S28A). Based on the base substitution frequency, these common sites were grouped into four clusters (Fig. S28B). In both the first and third clusters with high base substitution frequencies, the B subgenome (81.37% and 82.30%) was more represented than the A subgenome.

RNA editing functions by altering nucleotides in RNAs, and the distribution of RNA editing sites was analyzed to predict its functions. Most RNA editing sites occurred in exons (39.49%), followed by the 3’ UTR (30.75%, Fig. 5D). Over half of the mutations in the CDS regions were synonymous. Compared with the genome background, the RNA editing sites were significantly enriched in the CDS, 5’UTR, 3’UTR, and lncRNA regions (hypergeometric test, *P* = 4.40 × 10^−104^, 3.00 × 10^−9^, 1.26 × 10^−90^, 1.83 × 10^−27^, respectively). In particular, the frequency of RNA editing sites in lncRNA regions was 16.23-fold greater than the background (Fig. S29). The distribution of RNA editing sites was further compared between the two subgenomes. A higher proportion of RNA editing sites was in the lncRNA regions of the B subgenome (8.06%) than in those of the A subgenome (6.11%, *χ*^2^ test, *P* < 2.20 × 10^−16^; Fig. S30**)**. Additionally, the B subgenome had a significantly higher frequency of nonsynonymous substitution (*χ*^2^ test, *P* = 8.00 × 10^−11^**)**, but the proportion of RNA editing sites in the 3’UTR was significantly lower than that in the A subgenome (*χ*^2^ test, *P* = 1.68 × 10^−10^).

## Discussion

We reconstructed the common carp transcriptome by integrating data from PacBio full-length sequencing and Illumina RNA-seq. The improved annotation quality was evidenced by fewer BUSCO gene losses, a greater number of protein-coding genes, more complete gene structure, similar gene features, strong subgenome collinearity, and consistent expression levels among the different gene groups. An unusually high proportion (45.28%) of novel isoforms was detected in common carp, which is consistent with reports in zebrafish^28^ and goldfish^29^. These data indicate the widespread presence of unknown transcriptome diversity in Illumina RNA-seq-based annotations. Several factors have contributed to these missing genes or isoforms, including biases in library construction, sequence errors, short read alignments, and complex structural annotations^30^. PacBio sequencing technology has been widely used for AS identification in fish and has contributed to the understanding of the genetic basis of important traits. The advantage of long reads enabled the discovery of previously unidentified isoforms across multiple species. In rainbow trout, AS in the negative elongation factor C/D (*nelfcd*) and *titin* genes identified by PacBio sequencing may play key roles in regulating gene function, thereby affecting fish growth and muscle accretion^26^. In Nile tilapia, AS of the histone demethylase gene identified by PacBio sequencing was induced under high-temperature conditions, leading to female-to-male sex reversal^31^. PacBio sequencing provides additional advantages by avoiding PCR amplification. In the present study, the lncRNAs in the updated annotation were significantly longer than those previously reported, but there were no differences in mRNA length. This discrepancy may be attributable to biased PCR amplification of lncRNAs that may pose complex secondary structures. Even 245 lncRNAs that were not detected in any organ by Illumina RNA-seq were identified through PacBio sequencing. PacBio sequencing detected more AS events in UTR regions than did RNA-seq, highlighting the difference in coverage between the two methods. According to these data, previous annotations omitted a significant amount of information about noncoding regions, resulting in an insufficient understanding of their regulation. In goldfish^29^ and rainbow trout^26^, PacBio sequencing has also been used to reveal alternative polyadenylation patterns. In summary, the large number of novel genes and isoforms in this updated annotation not only offers valuable resources for genetic improvement of economically relevant traits but also highlights the significant advantages of PacBio sequencing in aquaculture transcriptome profiling.

Subgenome dominance has been widely observed in polyploids formed by WGD. Subgenome dominance of protein-coding genes has been detected in several allopolyploids^4,6,16,32^ as well as a few auto-polyploids^33^. In polyploid plants, differences in RNA structure^12^, protein translation efficiency^13^, and small RNAs^10^ have been revealed. Bias in the number of tRNAs and rRNAs among subgenomes has also been observed in cyprinid fish. However, except for protein-coding genes, it remains unclear whether other transcriptional and post-transcriptional events exhibit subgenome dominance in common carp. Therefore, this study identified AS, lncRNAs, circRNAs, and RNA editing across nine organs and conducted comparisons between the A and B subgenomes of common carp. The results revealed that all four types of transcriptional or post-transcriptional events occurred more frequently in the B subgenome. To eliminate the effects of homoeologous chromosome length, gene distribution, and tissue specificity, we conducted validations among homologous genes and across the nine organs. The results demonstrated that the B subgenome presented significantly greater numbers of AS events, circRNAs, lncRNAs, and RNA editing sites than did the A subgenome across all nine examined organs. This finding stands in marked contrast to observations in polyploid plants, where the dominant subgenome can vary between organs^7^. However, an increase in the number of transcriptional and post-transcriptional events did not necessarily correlate with a substantial increase in expression levels. We discovered that the average number of AS events, or RNA editing sites per gene was comparable in the A and B subgenomes. Similarly, no significant differences were detected in the expression levels of lncRNAs or circRNAs between the two subgenomes. Furthermore, the tissue specificities of these four transcriptional and post-transcriptional events were also found to be similar across the two subgenomes. These results suggested that the more transcriptional/post-transcriptional events in the B subgenome manifested primarily through the involvement of a greater number of genes, rather than through increased transcriptional intensity. This finding stands in significant contrast to the subgenome dominance observed in the expression levels of protein-coding genes^4–6,15^. Variations in the chromatin state may play crucial roles in the differential expression of protein-coding genes across subgenomes. In hexaploid wheat, chromosomes from different subgenomes exhibit relative independence and interact with each other through subgenome-biased transposons, thus participating in gene expression regulation^34^. Our previous study on common carp also found that differences in chromatin accessibility and DNA methylation influence gene expression levels^35^. The observed higher frequency, rather than increased abundance, of these four transcriptional or post-transcriptional events suggested the existence of an additional regulatory common to both subgenomes in common carp.

Several studies have been conducted on the origins of subgenome dominance. According to the general consensus, subgenome dominance is the result of TE-dominated differences in ancestral sequences and epigenetic modification levels^6,36^. This assumption, however, still faces many challenges. Allotetraploid common carp and goldfish exhibit parallel subgenome structures, which are characterized by similar transposon divergence and contents, better synteny levels, and subgenome dominance^4^. There is also evidence that differences in transposons are insufficient to initiate subgenome dominance in synthesized *Brassica* allotetraploids^37^. Therefore, it is more likely that differences in regulatory elements such as promoters and binding sites, rather than transposons, are responsible for subgenome dominance in common carp. In our study, the potential genomic and mRNA targets of lncRNAs were significantly differentially distributed between the two subgenomes, with an unexpectedly greater proportion of lncRNAs in the A subgenome interacting with the B subgenome. The expression patterns of circRNAs also highlight the importance of regulatory elements. The length of flanking introns, number of reverse complementary matches, and distribution of transposons are considered crucial factors in promoting the formation of circRNAs^38,39^. The equivalent expression levels of circRNAs corresponded to the similar contents of these regulatory elements between the two subgenomes of common carp. Additionally, there was a significant overlap between homoeologous genes that underwent these transcriptional and post-transcriptional events, which can also be attributed to the conservation of regulatory elements. Notably, the base substitution frequency of RNA editing sites was significantly higher in the B subgenome than in the A subgenome. This differs from other transcriptional events and is more closely related to the expression patterns of protein-coding genes. It can be explained by a single regulatory system inherited from the ancestor of the B subgenome and the preference of this system for various regulatory elements. To verify this assumption, further evolutionary and experimental studies are needed. In light of these data, we propose the importance of regulatory elements in subgenome dominance.

WGD provides a powerful genetic foundation for evolution, but also introduces redundancy that can burden organisms. Subgenome dominance and subsequently biased fractionation are thought to be key drivers of rediploidization. Traditionally, low-expressed genes faced more relaxed selective pressure and were more prone to loss or functional divergence. However, divergent gene expression, symmetric purifying selection, and unbiased gene loss have been detected simultaneously in several *Cyprinidae* species^4,6^. The distinctive subgenome dominance pattern observed in this study may be one of the important contributing factors to this phenomenon. The equivalent abundance of transcriptional events can correspond to comparable selective pressure and gene loss probabilities between the two subgenomes, whereas differences in frequency may contribute to subgenome functional differentiation. Indeed, subgenome dominance in common carp can still lead to divergent gene functions, as in ancient and recurrent WGD events^33,40^. We found that genes with AS in the A subgenome were enriched in the substance exchange process. RNA editing in the A subgenome was unusually active in the intestine, the primary organ for substance exchange in vivo. Similarly, parallel subgenome structure and divergent gene expression were also observed in goldfish. More mutations linked to morphological phenotypes were found in the S-subgenome of goldfish^41^, whereas the L-subgenome displayed a preferential function in neuron development compared with the S-subgenome in mesenchymal cells^32^. However, there is a lack of sufficient evidence for functional hypotheses regarding the particular subgenome dominance pattern observed in common carp. Further comparative studies in various natural or synthetic polyploids are needed to elucidate the mechanisms underlying the observed subgenome dominance patterns and their functional implications. Additionally, we found that a substantial number of GO terms related to immune and stimulus response pathways were enriched in multiple transcriptional or post-transcriptional events in both subgenomes. This functional redundancy not only suggests that the complex immune response operates at multiple transcriptional and post-transcriptional levels but also may contribute to the high adaptability of common carp to biotic and abiotic stresses.

## Methods

### Full-length transcriptome sequencing of common carp

Healthy one-year-old common carp individuals were cultivated at the hatchery station of the Chinese Academy of Fishery Sciences (Beijing, China). Nine organs (brain, gill, heart, intestine, kidney, liver, muscle, skin and spleen) were collected from six individuals. For each organ, total RNA was isolated via the FastPure Cell/Tissue Total RNA Isolation Kit V2 (Vazyme, China). Then, 1 µg of RNA per organ was pooled together to construct three single-molecule real-time (SMRT) libraries (1–2 kb, 2–3 kb, and 3–6 kb), which were sequenced on the PacBio RS II platform (PacBio, USA) via P6–C4 chemistry. SMRT Link v5.1^42^ was used to generate CCS reads with default parameters. Sequencing errors were corrected by Pilon v1.23^43^ with previously reported Illumina RNA-seq reads^4^ from the nine organs described above, with three iterations.

### Reannotation of common carp transcriptome

The common carp transcriptome was assembled using PacBio CCS reads and Illumina RNA-seq reads. First, the PacBio reads were aligned to the common carp reference genome (NCBI RefSeq assembly: ASM1834038v1) using GMAP v2020-06-30^44^ with the -f 3 parameter. Redundant full-length transcripts were removed from the generated transcriptome annotation using the cDNA-Cupcake (ToFU) pipeline v14.2.0^45^ with a minimum coverage of 85% and a minimum identity of 90%. Second, 27 RNA-seq data of nine organs described above^4^ were aligned to the reference genome using HISAT2 v2.2.1^46^ with default parameters. StringTie v1.3.3b^47^ was then used to generate transcript models. Afterward, the transcripts generated from the CCS reads and Illumina reads were merged into a nonredundant transcriptome annotation set guided by the NCBI genome reference annotation (GCF_018340385.1) using GffCompare v0.12.6^48^. Genes longer than 500 kb were reannotated via StringTie with the parameters -j 2 -f 0.05 -c 2. The final transcriptome annotation file was then imported into StringTie. Transcripts per million (TPM) values were calculated to quantify gene and transcript expression levels.

TransDecoder v5.5.0^49^ was used to predict protein sequences with a predicted length of more than 100 amino acids. The predicted protein sequences were then aligned to the Swiss-Prot database^50^ using BLASTP v2.7.1+ with the parameters -k 1 -evalue 0.00001. Gene Ontology (GO) terms were assigned based on the alignments. Additionally, KEGG pathways for each gene were annotated using the KOBAS online tool^51^. Furthermore, the accuracy of the updated annotation was evaluated by comparing the proportion of homoeologous genes in closely related species. Homoeologous genes were generated by aligning the longest protein of each gene of common carp against the representative protein sequences of *Paracanthobrama guichenoti*, *Puntius tetrazona* and zebrafish, respectively, via BLASTP with the parameters -evalue 0.00001 -max_target_seqs 1. Collinearity analysis of the two subgenomes in common carp was performed using MCScanX^52^ with a minimum of five homoeologs, and synteny was visualized using TBtools v1.095^53^. Additionally, gene completeness was assessed using BUSCO v5.1.3^54^ with the *Actinopterygii* dataset.

### Identification of alternative splicing events

Genes with multiple isoforms were subject to AS. To analyze the tissue specificity of AS, UpSetR v1.4.0^55^ was used to visualize the intersections of expressed isoforms. GO and KEGG enrichment analysis for genes that underwent AS were conducted using TBtools v1.095^53^. GO or KEGG terms were considered significantly enriched when the adjusted *P* value was ≤0.05. Additionally, the effectiveness of different sequencing methods for detecting AS events was evaluated. Astalavista v3.2^56^ was employed to compare the distributions of AS events detected by the two methods.

### Expression and functional analysis of lncRNAs

Transcripts with a length greater than 200 bp and without a predicted open reading frame were submitted to FEELnc v0.2.1^57^. lncRNAs were identified based on the features extracted from random intergenic sequences and known mRNAs with conserved protein domains. The tissue specificity of lncRNA expression was measured via Shannon entropy as follows^58^:

$$H\left({x}_{i}\right)=-{\sum}_{n=1}^{m}P\left({x}_{i}\right){\log }_{2}\left[P\left({x}_{i}\right)\right]$$

where m is equal to the number of organs, ${x}_{i}$ is the expression level of lncRNA $i$ in the organ, and the probability of lncRNA $i$ expression $P\left({x}_{i}\right)$ in the $n$ organ is determined by dividing the TPM value in the organ by the sum of the TPM values in all organs.

For lncRNAs originating from protein-coding genes, we examined the GO functions of the lncRNA host genes using TBtools. The functions of lncRNAs have been investigated in two ways. First, Triplexator v1.3.2^59^ was used to identify potential interactions between lncRNAs and genomic DNA using the following parameters: -l 20 -e 5 -fr on -mrl 7 -mrp 3 -dc 5 -of 1 -po -rm 3 -p 3 -dd 1. These targeted genomic loci were then annotated using ChIPseeker v1.26.2^60^. Second, to mine the potential interactions between lncRNAs and mRNAs, BLASTN was used to build lncRNA‒mRNA pairs based on reverse complementary matches with at least 20 bp and 90% identity. To assess the potential functions of these interactions, Euclidean distances and correlation coefficients were calculated for the expression levels of lncRNA‒mRNA pairs. Euclidean distances of 5.10 and correlation coefficients of 0.667 were used as the thresholds, as reported in a previous study^4^.

### Characterization of circular RNAs

CIRCexplorer2 v2.2.7^38^ was employed to identify circRNAs by detecting back-splicing reads. The Illumina RNA-seq reads were aligned to the reference genome using TopHat2 v2.0.12 with the parameters -m 2 -a 6 -microexon-search, guided by the updated annotation^61^. The unaligned reads were subsequently realigned by TopHat2 with the parameters --bowtie-1 --no-coverage-search to generate the fusion junction alignments. CIRCexplorer2 was then used to parse the back-splicing junction and annotate splicing reads based on the updated gene annotations. To minimize the false-positive rate, only candidates with at least two back-splicing reads were considered circRNAs. The expression levels of circRNAs in each organ were quantified via normalization of the back-splicing read counts to the junction reads number per million mapped reads. GO enrichment analysis was conducted to examine the functions of circRNA host genes using TBtools.

### Detection and analysis of RNA editing in nine organs

The Illumina RNA-Seq reads of nine organs were aligned to the common carp reference genome using STAR v2.5.3a^62^ with the following parameters: --sjdbOverhang 149 --outSAMattrIHstart 0 --outFilterMismatchNmax 4 --alignIntronMax 20000. To distinguish RNA editing from genomic SNPs, the resequencing reads of common carp (SRA accession: SRR13247176)^4^ were aligned to the reference genome using BWA v0.7.17^63^ with default parameters. Resequencing reads and RNA-seq reads with multiple hits were excluded from the analysis. The REDItoolDnaRna.py script of REDItools v1.3^64^ was employed to detect RNA editing candidates. Candidate sites were further filtered using the selectPositions.py script with the following criteria: both coverage of RNA-seq and DNA-seq ≥10, variation frequency ≥ 0.1, nonvariation for DNA-seq reads, and unique mutation. Only RNA editing sites identified in at least two RNA-seq replicates were included. ANNOVAR v20200607^65^ was used to determine changes in protein-coding sequences resulting from RNA editing events.

### Statistics and reproducibility

Details of statistical analyses used in each analysis were described in the Methods and Results section. R 4.2.3 (https://www.r-project.org/) was used for statistical analyses.

### Reporting summary

Further information on research design is available in the Nature Portfolio Reporting Summary linked to this article.

## Declarations

### Ethics approval

The study was approved by the Animal Care and Use Committee of the Chinese Academy of Fishery Sciences (ACUC-CAFS, Permit Number: ACUC-CAFS-20180034), and conducted following the recommendations of the Care and Use of Animals for Scientific Purposes established by ACUC-CAFS. Before collecting the organs, all fishes were euthanized in MS222 solution.

## Supplementary information

Supplementary Information

Description of Additional Supplementary File

Supplementary Data

Reporting Summary

## Data Availability

Supplementary Tables 1–13 in this study are provided in the Supplementary data zip file. The PacBio Iso-seq data were deposited in the NCBI SRA database under accession number PRJNA752470. The RNA-seq data of nine common carp organs were downloaded from the NCBI SRA database under accession number PRJNA684670. The improved transcriptome annotations and data supporting the results reported in the manuscript are available in Figshare (10.6084/m9.figshare.25283650)^66^.

## Code availability

No custom code was developed for this study.
