# Supplementary material for: An improved transcriptome annotation reveals asymmetric expression and distinct regulation patterns in allotetraploid common carp
Source: Commun Biol. 2024 Nov 20;7:1542. doi: 10.1038/s42003-024-07177-3 (PMC11579021; doi:10.1038/s42003-024-07177-3)
Supplement: Supplementary file 1 — Supplementary Information [file 42003_2024_7177_MOESM1_ESM.pdf]

Supplementary Figures

Fig. S1. Read length distribution of PacBio SMRT sequencing data..3

Fig. S2. Summary of transcriptional and post-transcriptional events identified in common carp 4

Fig. S3. Comparison of length distributions between transcriptome annotations of common carp and zebrafish 5

Fig. S4. Sequence features of isoforms in different annotation groups..6

Fig. S5. Expression profiles of isoforms in different annotation groups...7

Fig. S6. Protein sequence identity and expression profiles of isoforms in different annotation groups..8

Fig. S7. Chromosome distribution of genes with alternative splicing events 9

Fig. S8. Intersection of homoeologous genes with alternative splicing between the A and B subgenomes...10

Fig. S9. Comparison of expression levels of genes with alternative splicing in the two subgenomes 11

Fig. S10. Statistics on the number of isoforms expressed in nine organs 12

Fig. S11. Summary of GO terms enriched in genes with alternative splicing in the A and B subgenomes...13

Fig. S12. Summary of alternative splicing events identified by Illumina and SMRT sequencing..14

Fig. S13. Length distribution of lncRNAs in the updated annotation...15

Fig. S14. Schematic of collinearity between lncRNAs originating from the A and B subgenomes in common carp...16

Fig. S15. Comparison of expression patterns of lncRNAs and protein-coding mRNAs in nine organs...17

Fig. S16. Comparison of the lncRNA expression levels in the A and B subgenomes....................18

Fig. S17. Summary of GO terms enriched in lncRNA host genes in the A and B subgenomes......19

Fig. S18. Expression correlation and Euclidean distance of lncRNA–mRNA pairs in common carp 20

Fig. S19. Expression correlation and Euclidean distance of lncRNA–mRNA pairs in the A and B subgenomes...21

Fig. S20. Distribution of circRNAs in the two subgenomes of common carp 22
Fig. S21. Comparison of the sequence features of introns flanking circRNA in the A and B subgenomes 23
Fig. S22. Comparison of the percentage of flanking introns with various transposons in the A and B subgenomes 24
Fig. S23. Expression profiling of circRNAs in nine organs 25
Fig. S24. Number and base substitution frequency of various types of RNA editing sites 26
Fig. S25. Upset plot of RNA editing sites identified in nine organs 27
Fig. S26. Statistics on the number of RNA editing sites in the A and B subgenomes of common carp 28
Fig. S27. Base substitution frequency of RNA editing sites in homoeologous genes 29
Fig. S28. Comparison of base substitution frequency of RNA editing sites shared by nine organs in common carp 30
Fig. S29. Enrichment of RNA editing sites in various genetic elements 31
Fig. S30. Genome distribution of RNA editing sites in the A and B subgenomes 32

Fig. S1. Read length distribution of PacBio SMRT sequencing data

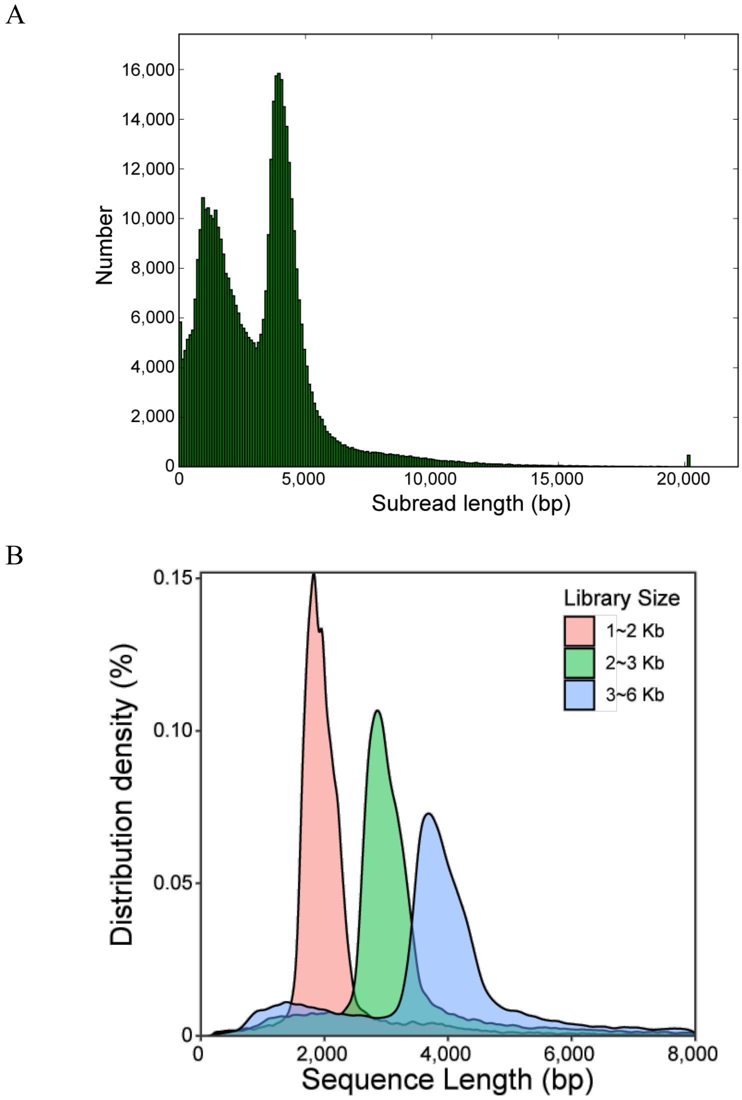

(A). Histogram plot of subread number grouped by length (bin = 100 bp). (B). Distribution density of ROI (Read of Insert) length for PacBio SMRT sequencing.

Fig. S2. Summary of transcriptional and post-transcriptional events identified in common carp

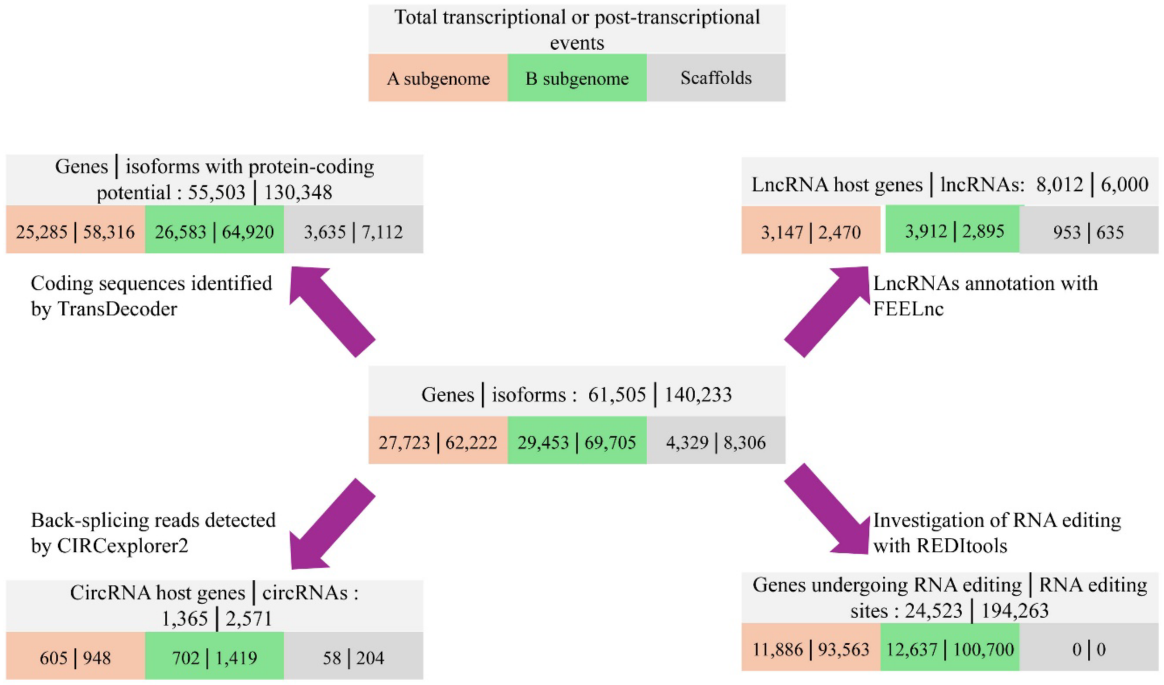

*: RNA editing events were not detected in the scaffold sequences due to issues with the identification pipeline.

Fig. S3. Comparison of length distributions between transcriptome annotations of common carp and zebrafish

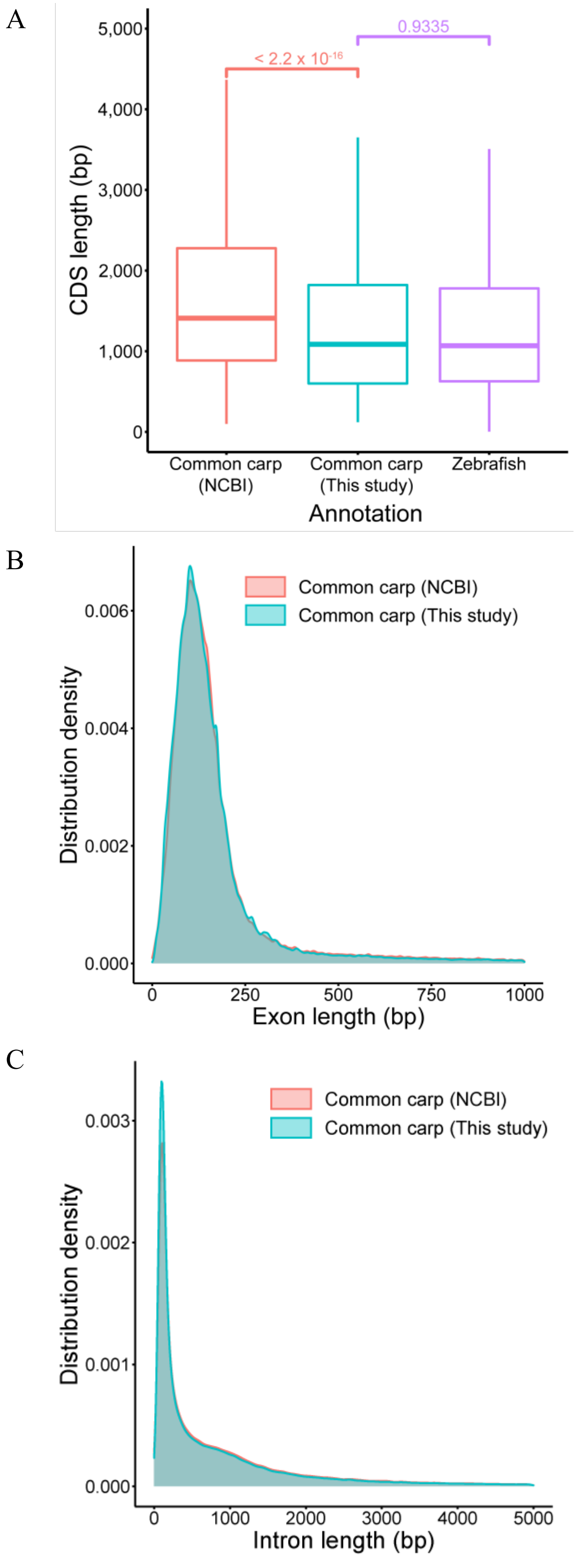

(A) Although CDS length of the updated annotation was lower than that of the NCBI reference annotation of common carp, it was equivalent to the CDS length of the zebrafish transcriptome annotation (Wilcoxon rank-sum test). The length of exon (B) and intron (C) did not show significantly difference between the updated annotation and the NCBI reference annotation (Wilcoxon rank-sum test).

Fig. S4. Sequence features of isoforms in different annotation groups

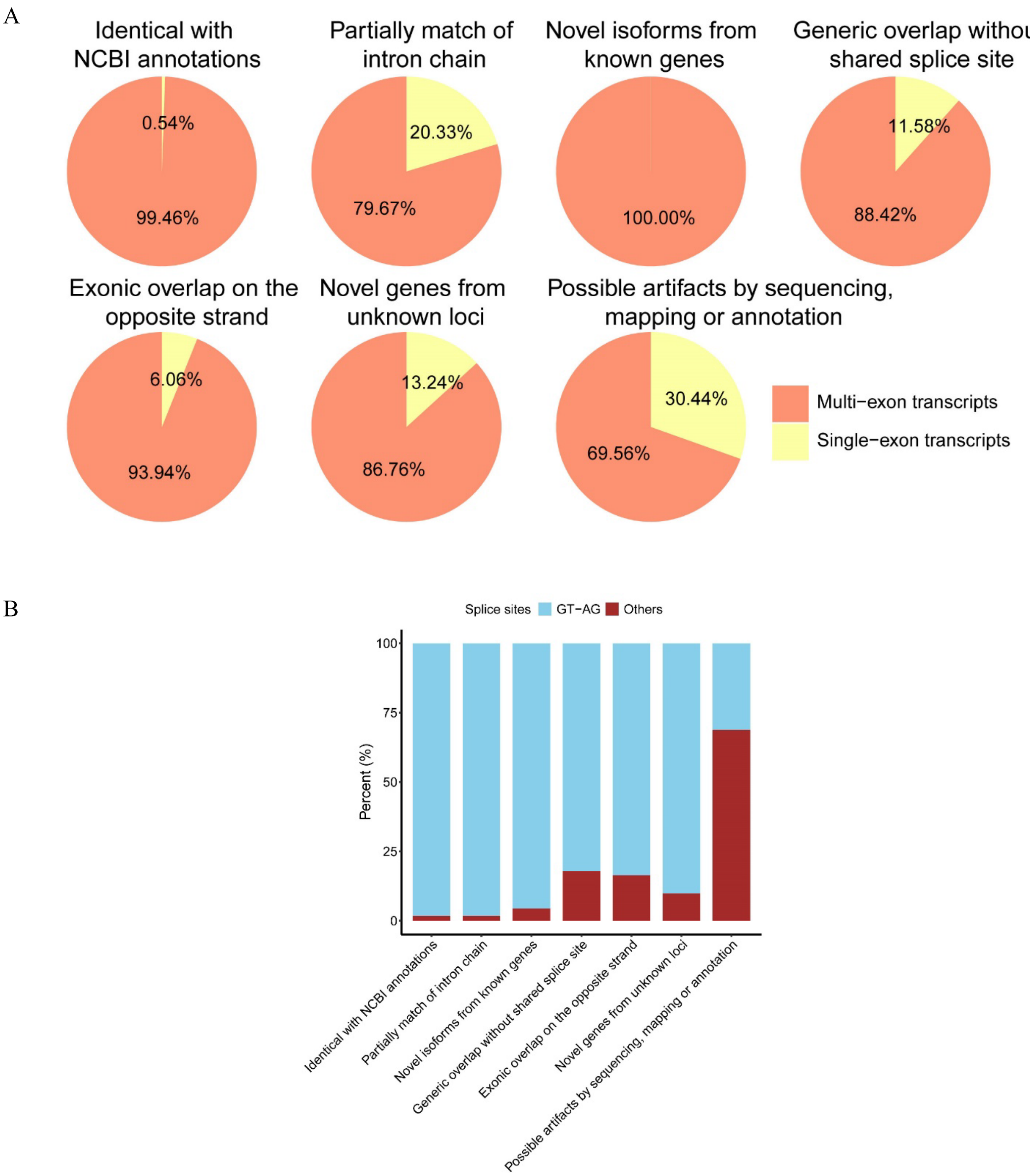

(A) The pie chart illustrated the classification of multi-exon and single-exon transcripts in different groups.
(B) The bar plot showed the percentage of splicing signal detect at intron sites.

Fig. S5. Expression profiles of isoforms in different annotation groups

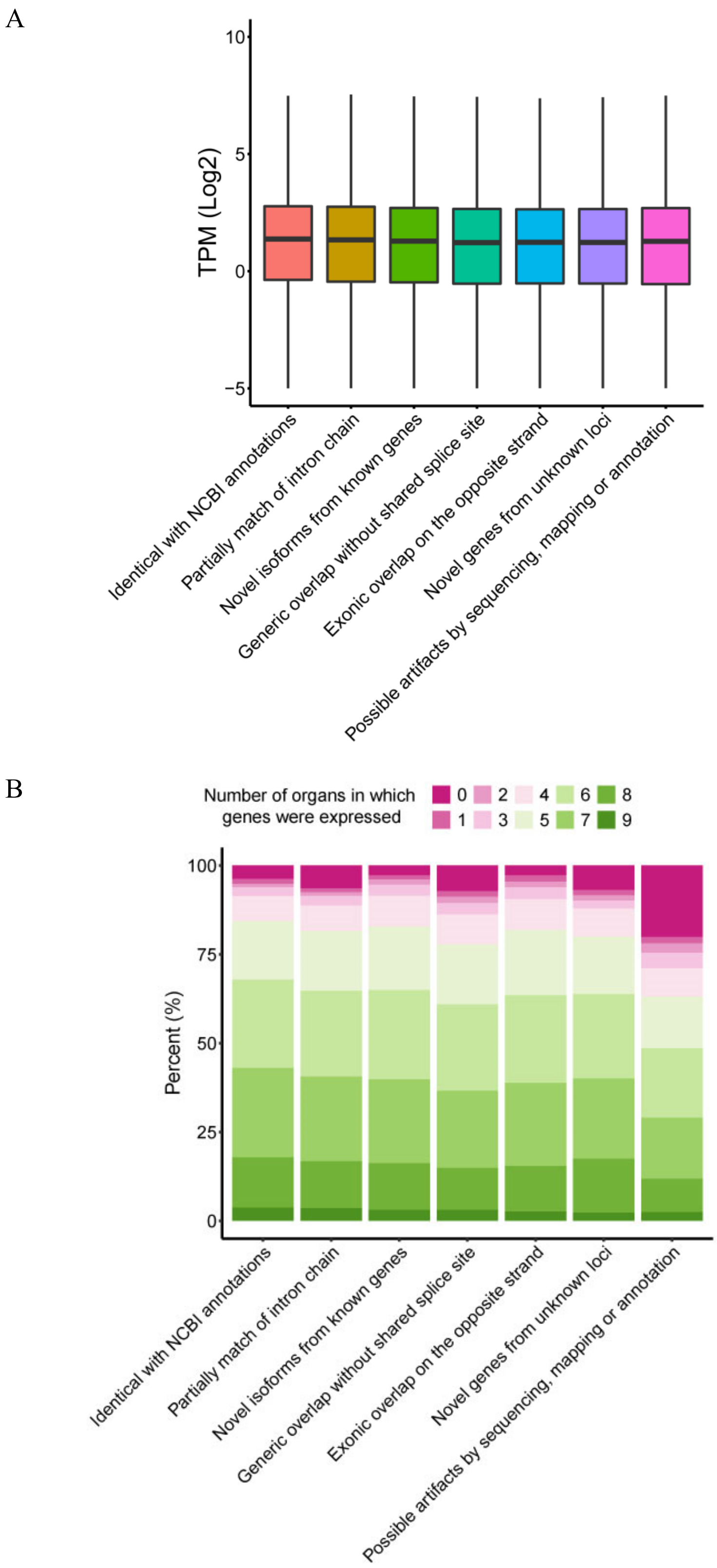

(A) The boxplot illustrated the expression levels of various isoforms grouped by different gene model matches. (B) The bar plot demonstrated the expression specificity of isoforms in different groups across nine organs.

Fig. S6. Protein sequence identity and expression profiles of isoforms in different annotation groups.

A

B

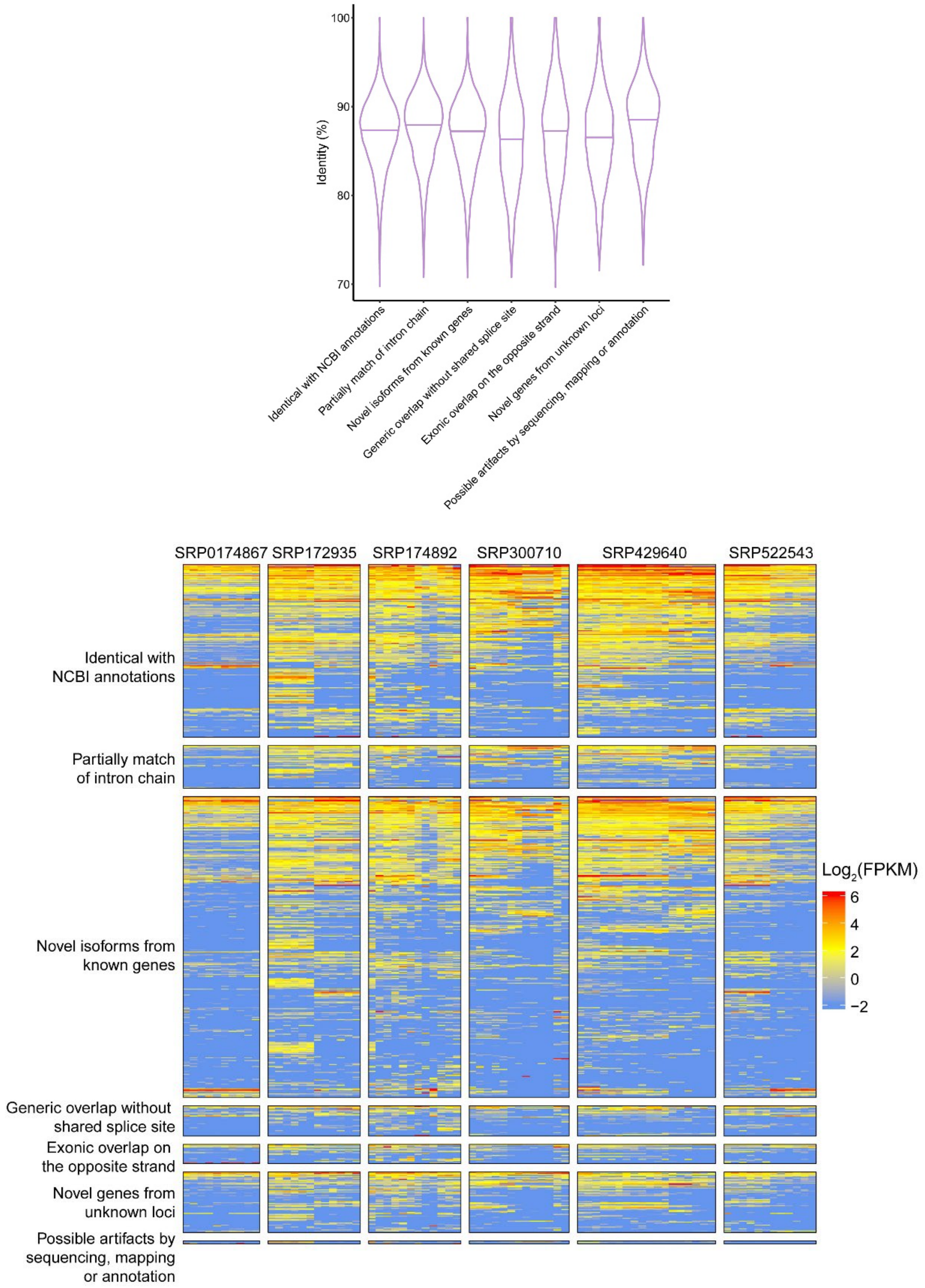

(A) The boxplot illustrated the sequence identity of various protein-coding isoforms in different groups with zebrafish proteins. (B) The heatmap showed the expression profiles of identified isoforms in various RNA-seq experiments (downloaded from the NCBI SRA database). Accessions were showed in the top panel).

Fig. S7. Chromosome distribution of genes with alternative splicing events

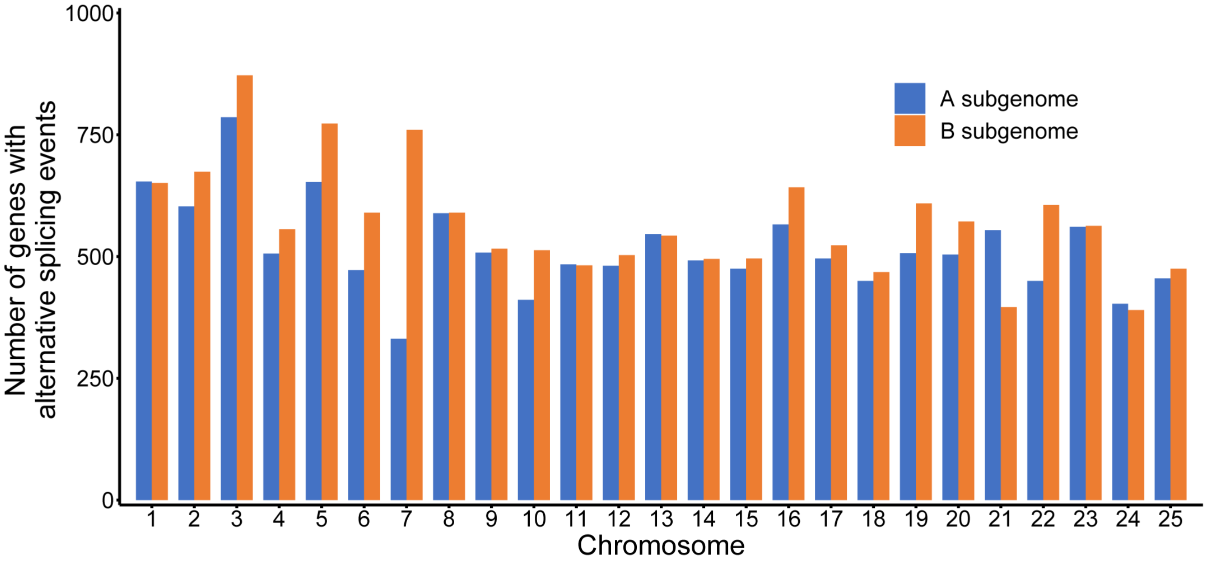

Fig. S8. Intersection of homoeologous genes with alternative splicing between the A and B subgenomes

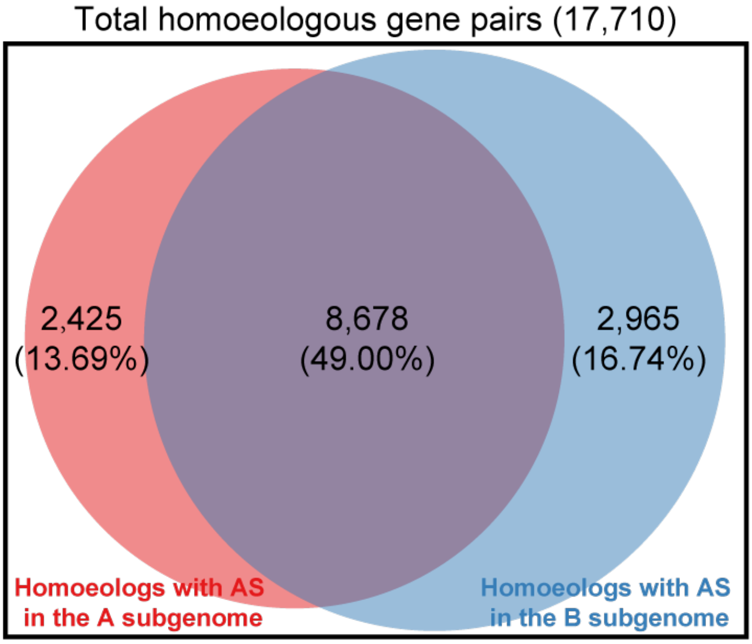

Fig. S9. Comparison of expression levels of genes with alternative splicing in the two subgenomes

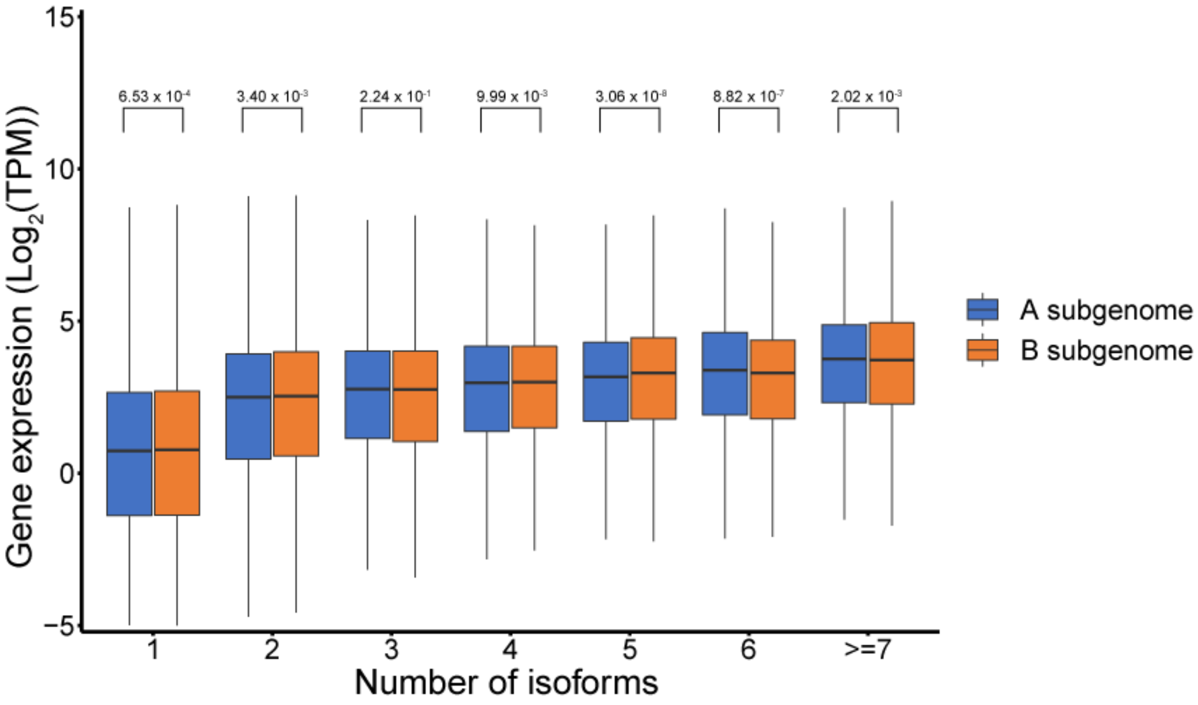

The boxplot showed expression levels of genes with varying numbers of isoforms in the A and B subgenomes. *P* values derived from Wilcoxon rank-sum test were annotated in the graph.

Fig. S10. Statistics on the number of isoforms expressed in nine organs

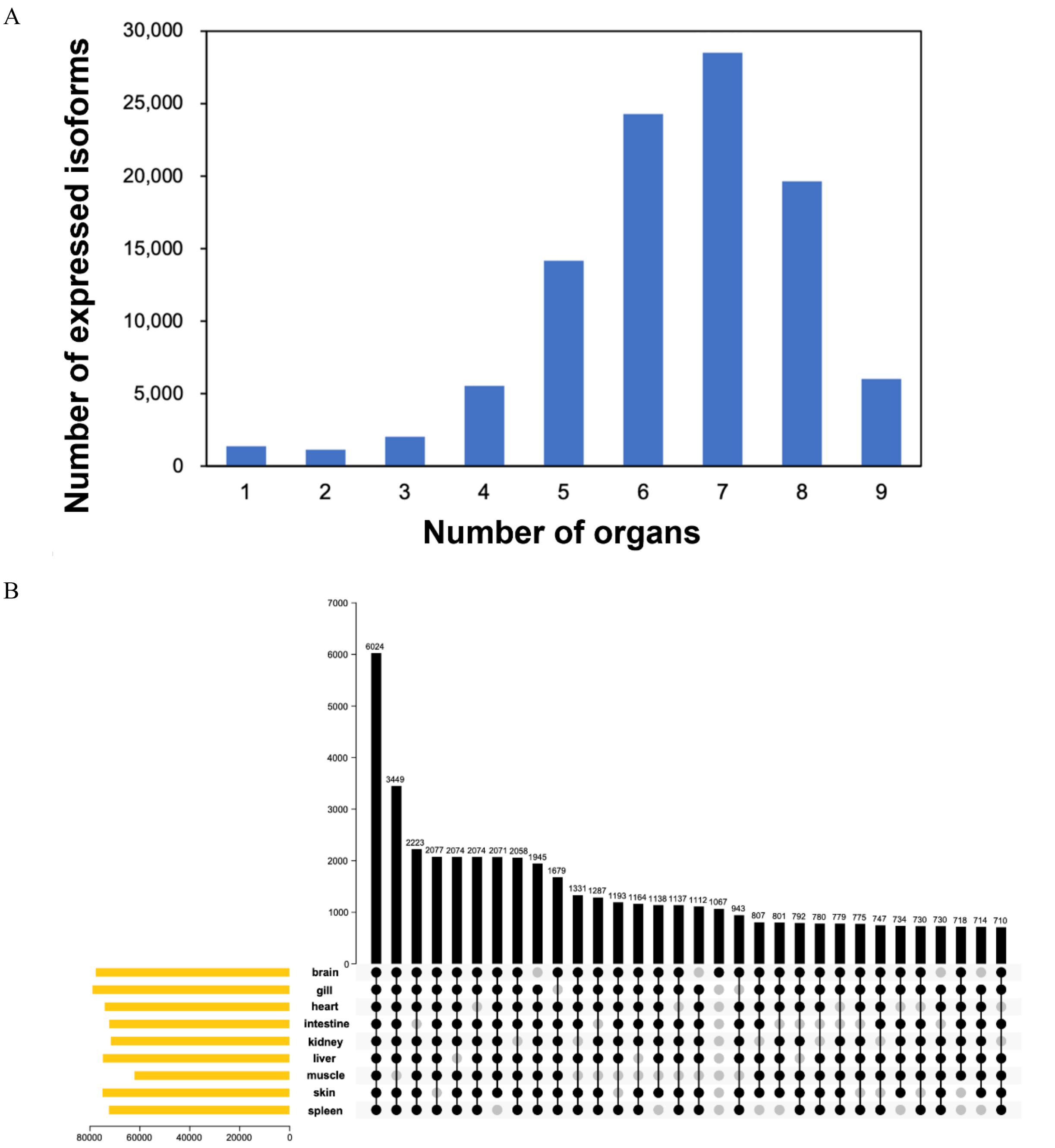

(A) Histogram of the number of isoforms expressed in different organs. X axis means the number of organs in where isoforms were expressed. (B) Upset plot depicting the number of unique and shared isoforms in each organ. The orange bars on the left indicated the total number of expressed isoforms in each organ. The set of isoforms shared between organs was represented by black dots connected by lines, and the number was displayed by the top vertically aligned bar plot.

Fig. S11. Summary of GO terms enriched in genes with alternative splicing in the A and B subgenomes

A

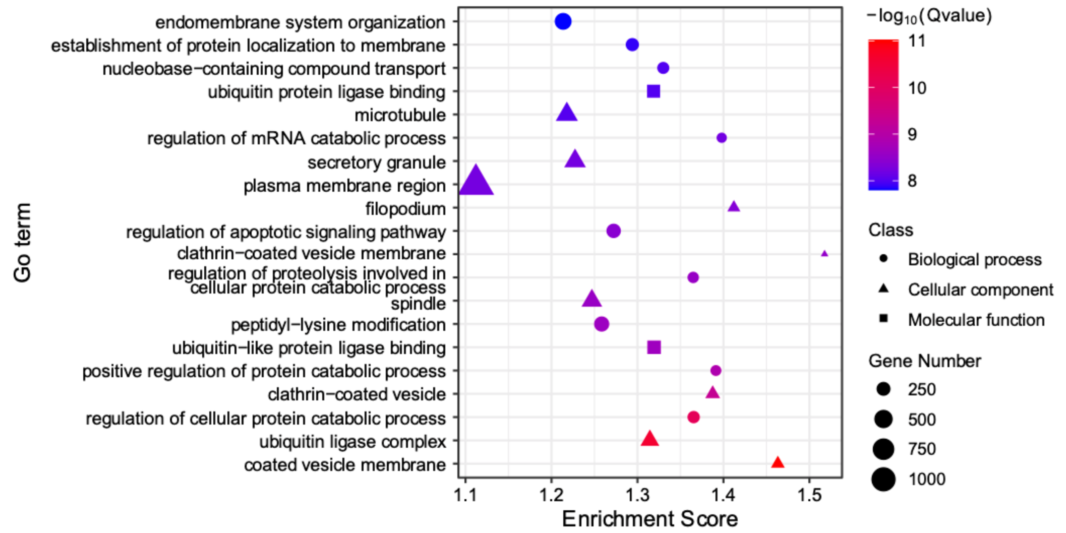

B

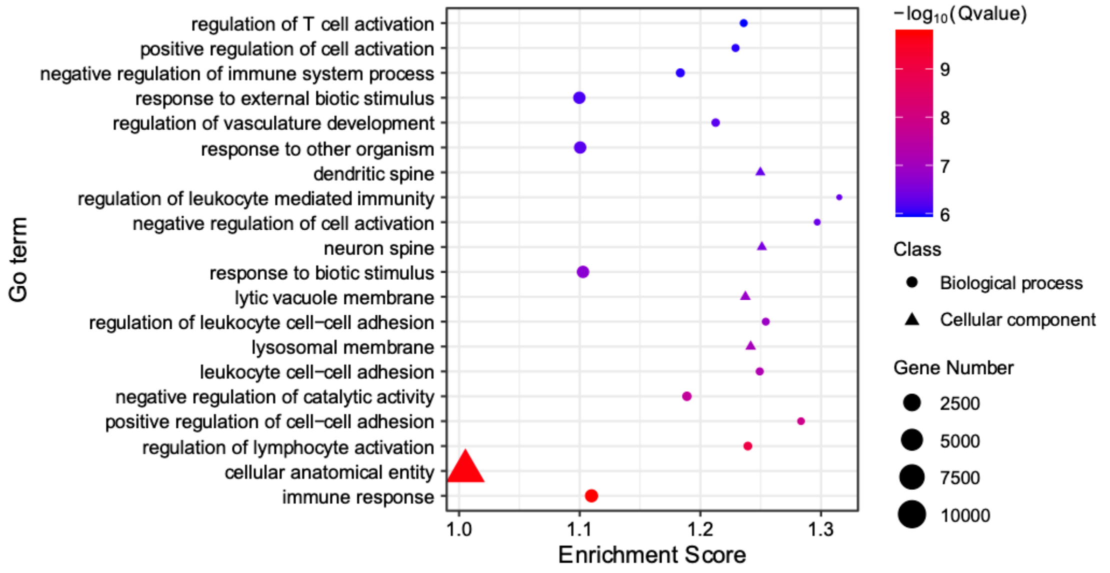

The specific (top 20) enriched GO terms associated with genes with AS in the A (A) and B (B) subgenomes. The Q value was defined as the *P* value corrected by the BH (Benjamini & Hochberg, 1995) method.

Fig. S12. Summary of alternative splicing events identified by Illumina and SMRT sequencing

A

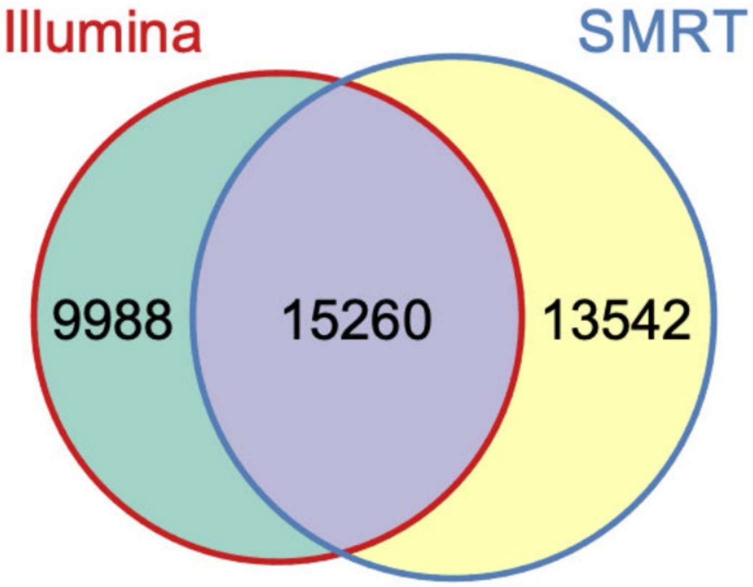

B

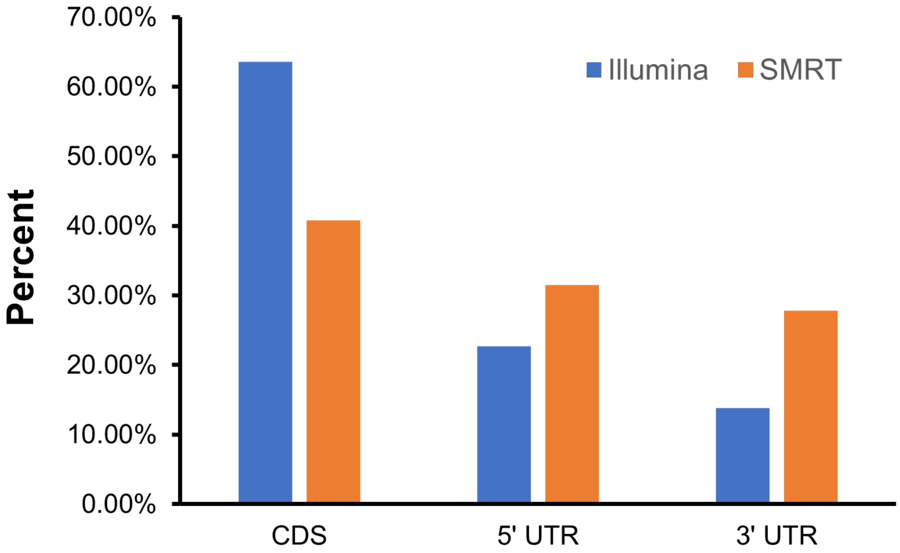

(A) Venn diagram showed the overlap of alternative splicing (AS) events identified by Illumina and SMRT sequencing. (B). The bar plot illustrated the positional distribution of AS events on transcripts, as identified by Illumina and SMRT sequencing methods.

Fig. S13. Length distribution of lncRNAs in the updated annotation

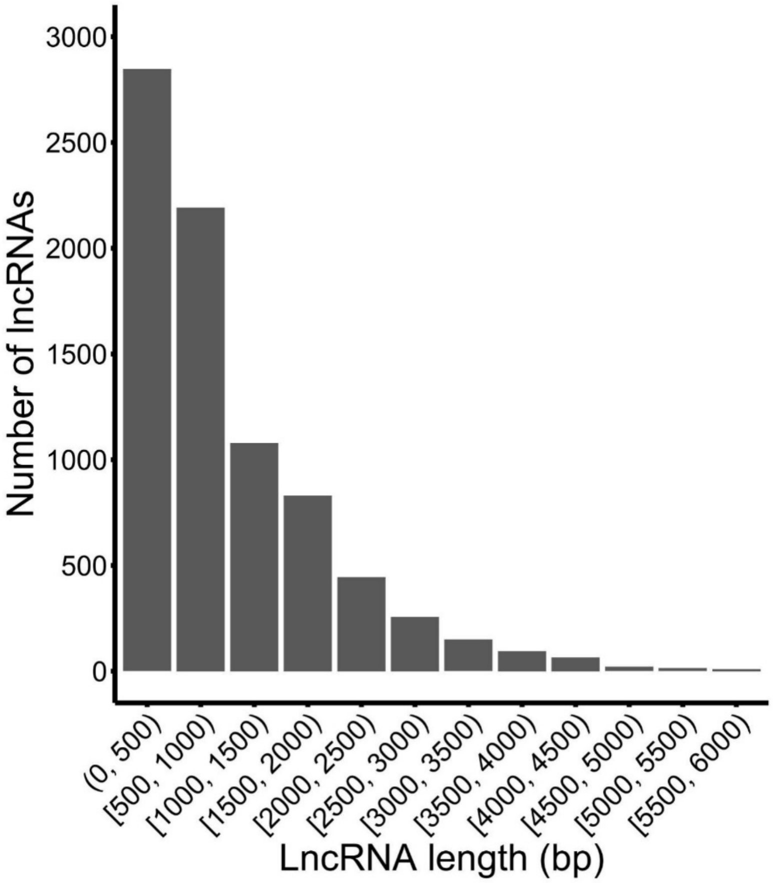

Fig. S14. Schematic of collinearity between lncRNAs originating from the A and B subgenomes in common carp

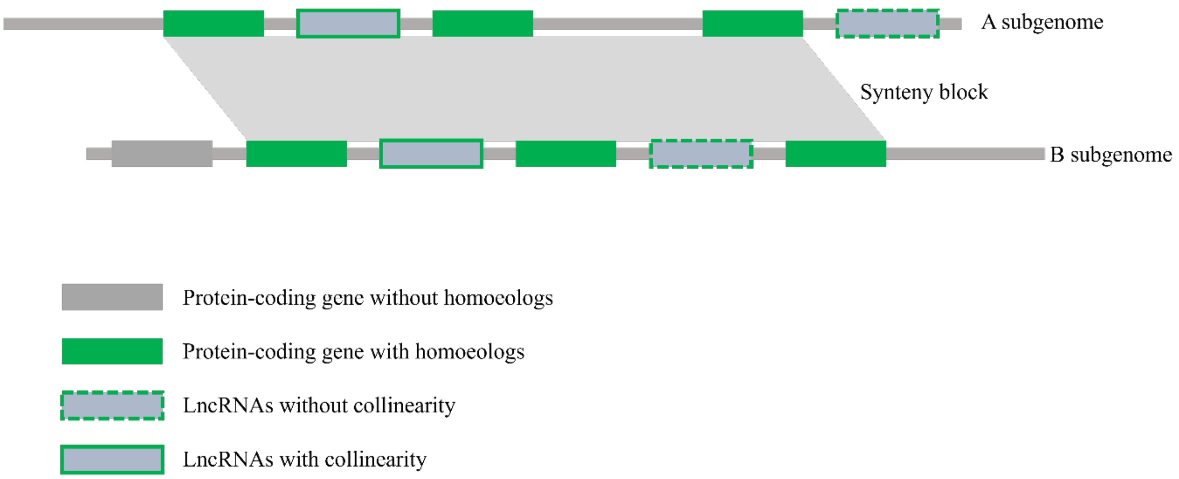

Fig. S15. Comparison of expression patterns of lncRNAs and protein-coding mRNAs in nine organs

A

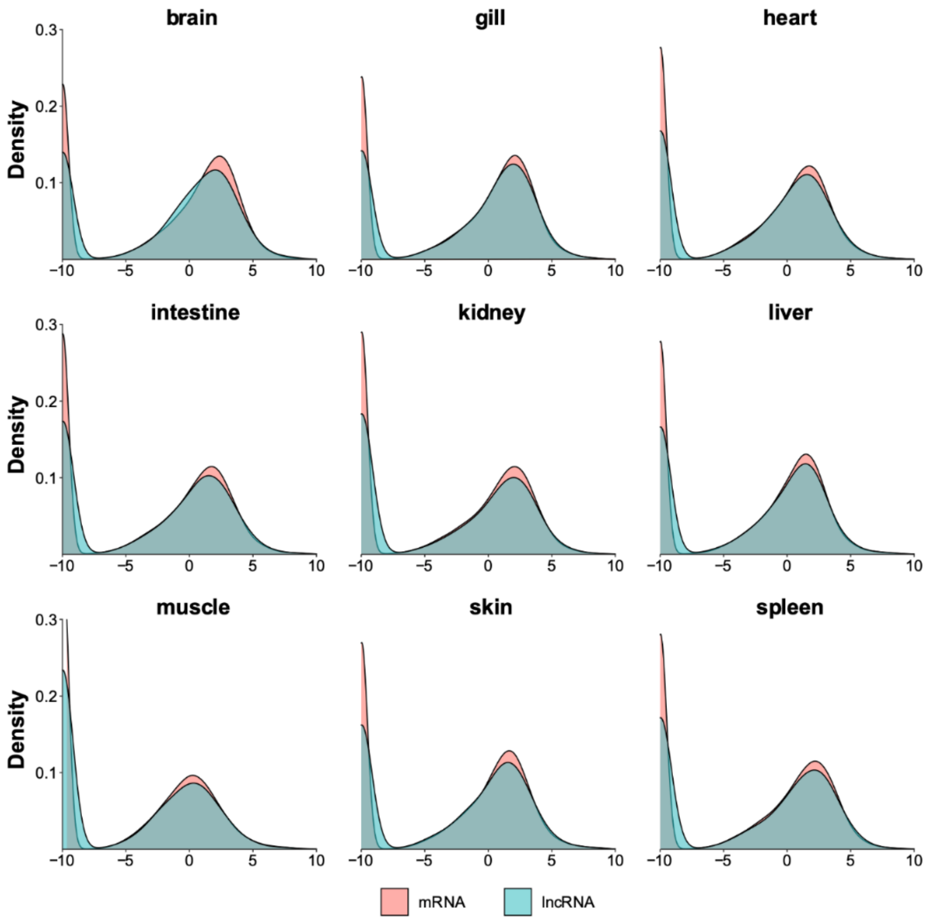

B

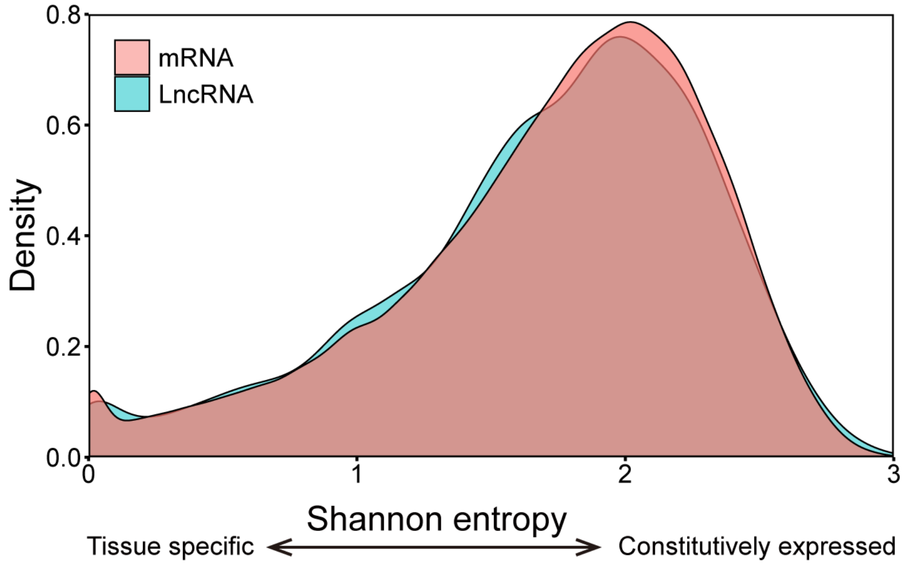

(A) The X-axis showed the TPM normalized by the Log2 function in R, and all values had been added 0.001 to eliminate missing data. LncRNA and mRNA showed similar expression levels in nine organs. (B). Density plot of Shannon entropy of lncRNA and mRNA with protein-coding potential. LncRNA and mRNA showed similar tissue specificity in common carp.

Fig. S16. Comparison of the lncRNA expression levels in the A and B subgenomes

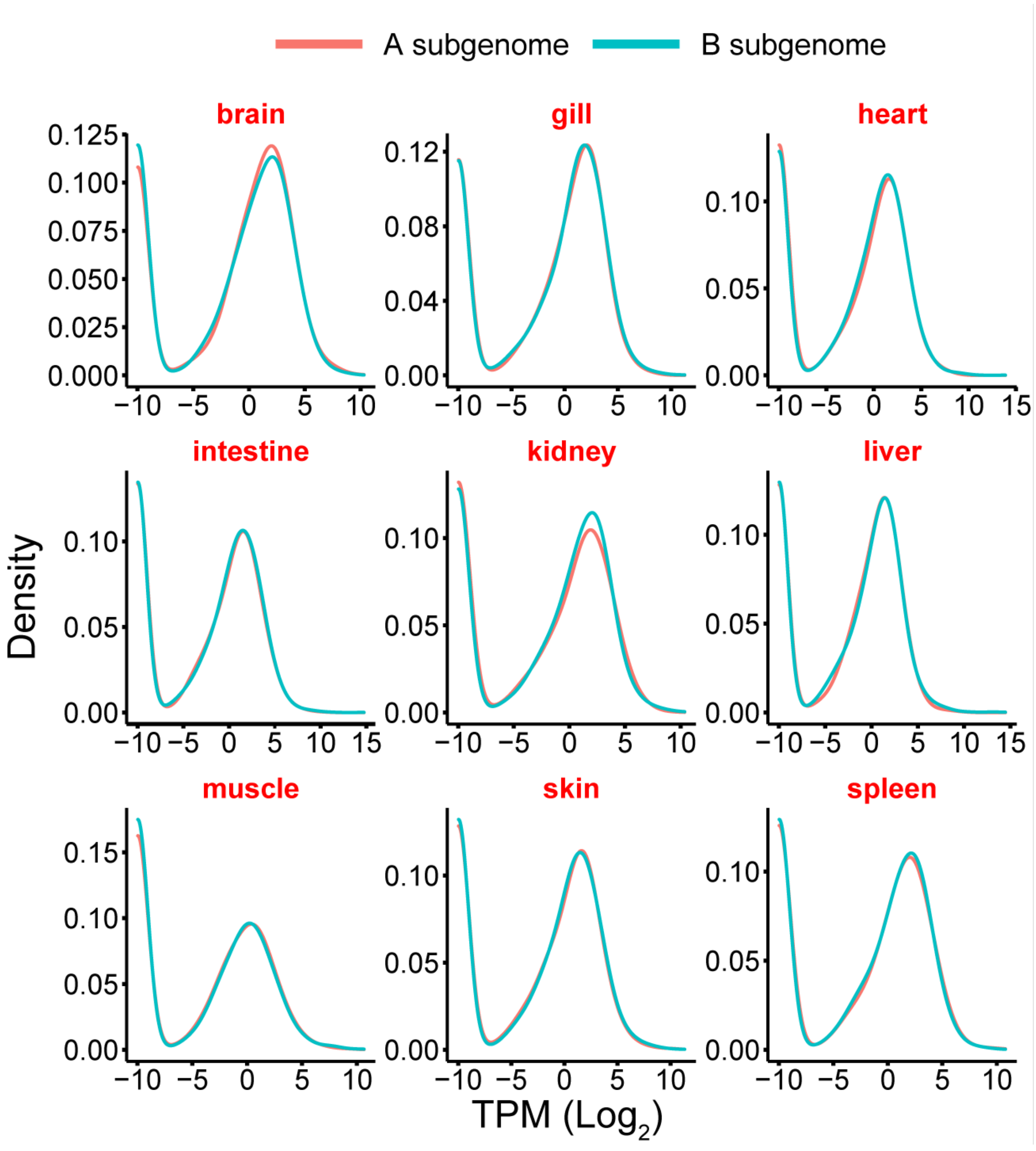

The X-axis showed the TPM normalized by the Log2 function in R, and all values had been added 0.001 to eliminate missing data. LncRNAs originating from the A and B subgenomes showed similar expression levels.

Fig. S17. Summary of GO terms enriched in lncRNA host genes in the A and B subgenomes

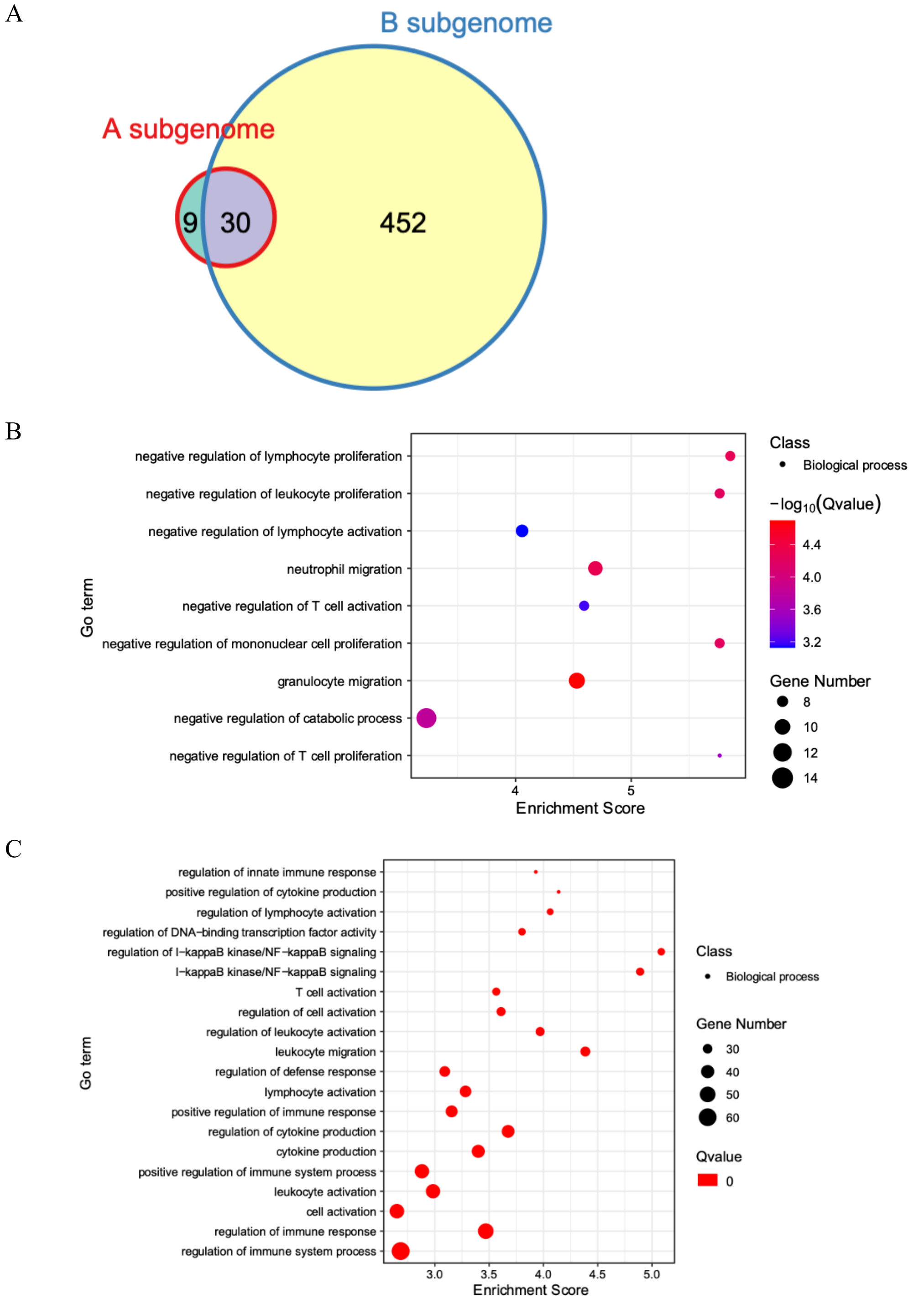

(A) Venn diagram of GO terms enriched in lncRNA host genes in the A and B subgenomes. Bubble plot showing GO terms only found in the A subgenome (B) and top twenty specific terms in the B subgenome (C). The Q value was defined as the *P* value corrected by the BH (Benjamini & Hochberg, 1995) method.

Fig. S18. Expression correlation and Euclidean distance of lncRNA–mRNA pairs in common carp

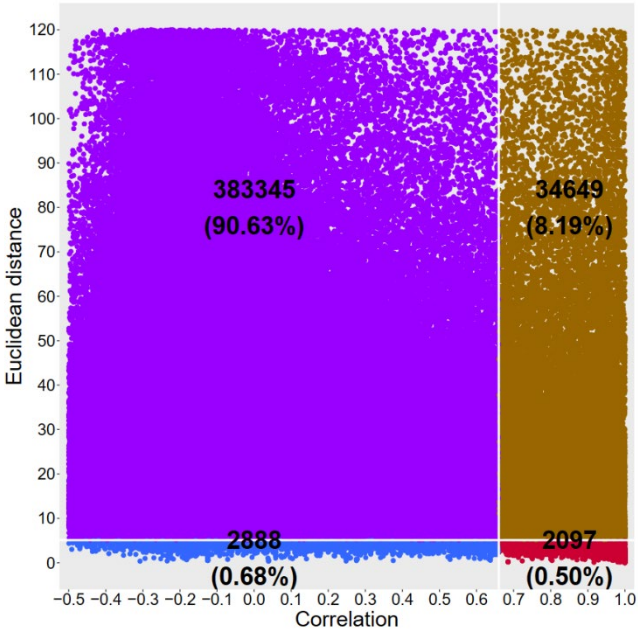

The scatterplot of the expression correlations (x-axis) and Euclidean distances (y-axis) of lncRNA-mRNA pairs. The figure was gapped by the 90th quantile of Euclidean distance and expression correlation of 0.667, and total pairs were classified into four groups. The top left (purple) showed lncRNA-mRNA pairs with high Euclidean distances (greater than or equal to the 10th Euclidean distance threshold) and low correlation (≤0.667); the bottom left (blue) showed with low Euclidean distance and low correlation; upper right (brown) showed with high Euclidean distance and high correlation; and lncRNA-mRNA pairs in the lower right (red) had low Euclidean distance and high correlation. Each box listed the number and percentage (in brackets) of lncRNA-mRNA pairs for each group. The majority of lncRNA-mRNA pairs (90.63%) had divergent expression patterns with large Euclidean distances and low correlations.

Fig. S19. Expression correlation and Euclidean distance of lncRNA–mRNA pairs in the A and B subgenomes

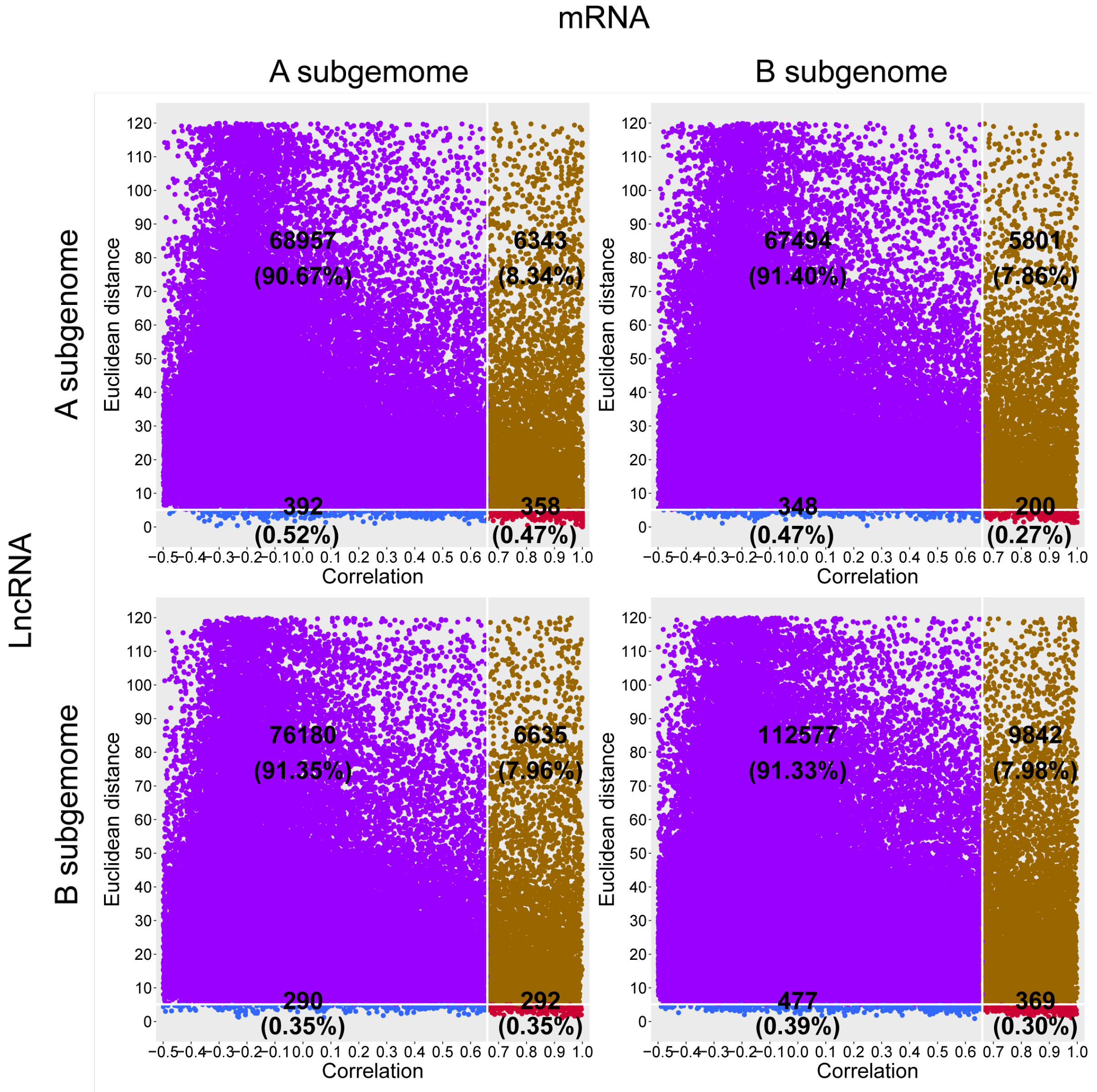

The scatterplot of the expression correlations (x-axis) and Euclidean distances (y-axis) of lncRNA-mRNA pairs. Top left: lncRNA (in the A subgenome) and mRNA (in the A subgenome), Top right: lncRNA (in the A subgenome) and mRNA (in the B subgenome), Bottom left: lncRNA (in the B subgenome) and mRNA (in the A subgenome), Bottom right: lncRNA (in the B subgenome) and mRNA (in the B subgenome).

Fig. S20. Distribution of circRNAs in the two subgenomes of common carp

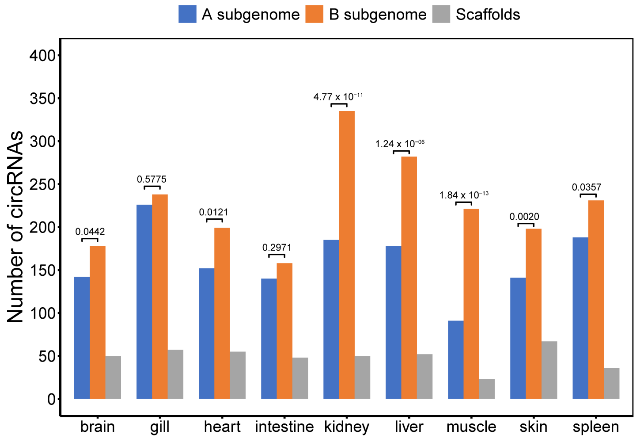

Number of circRNAs detected in nine organs. *P* values of chi-squared test were showed in the figure.

Fig. S21. Comparison of the sequence features of introns flanking circRNA in the A and B subgenomes

A

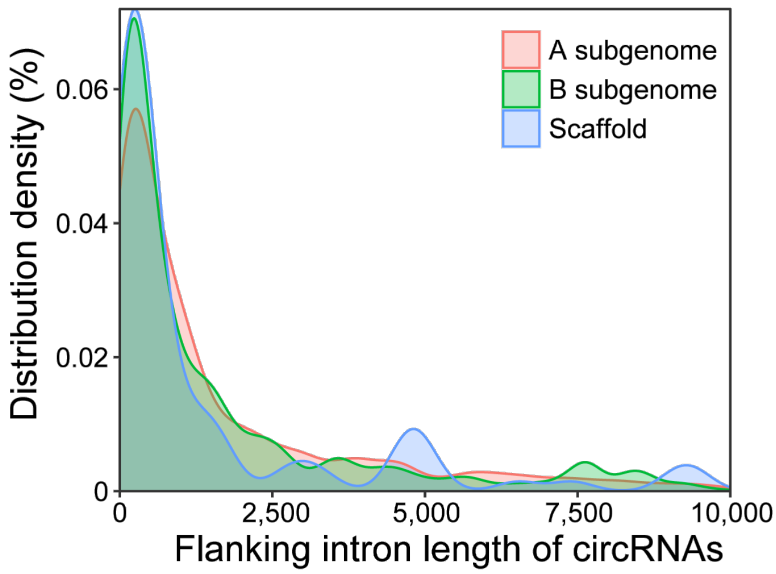

B

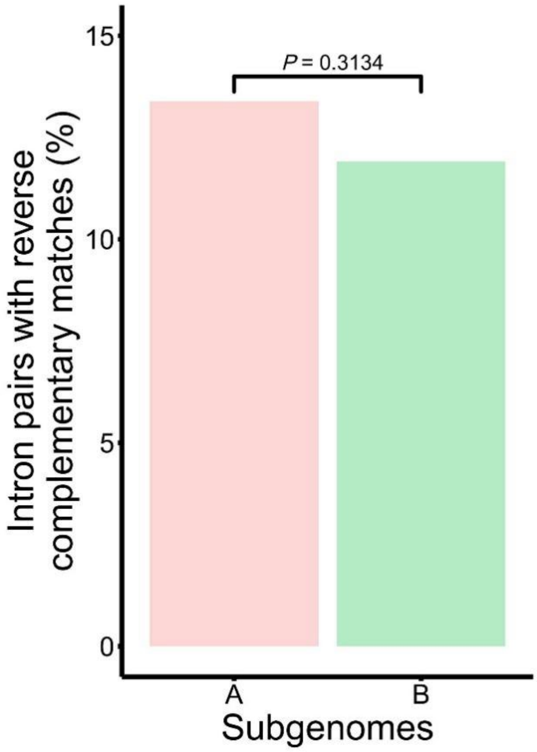

(A) Length distribution of introns flanking circRNA in A, B subgenomes and scaffolds. (B) Percentage of circRNAs that contain flanking intron pairs with reverse complementary matches. *P* Values for chi-squared test to test differences in percentage of circRNAs with reverse complementary matches across the A and B subgenomes.

Fig. S22. Comparison of the percentage of flanking introns with various transposons in the A and B subgenomes

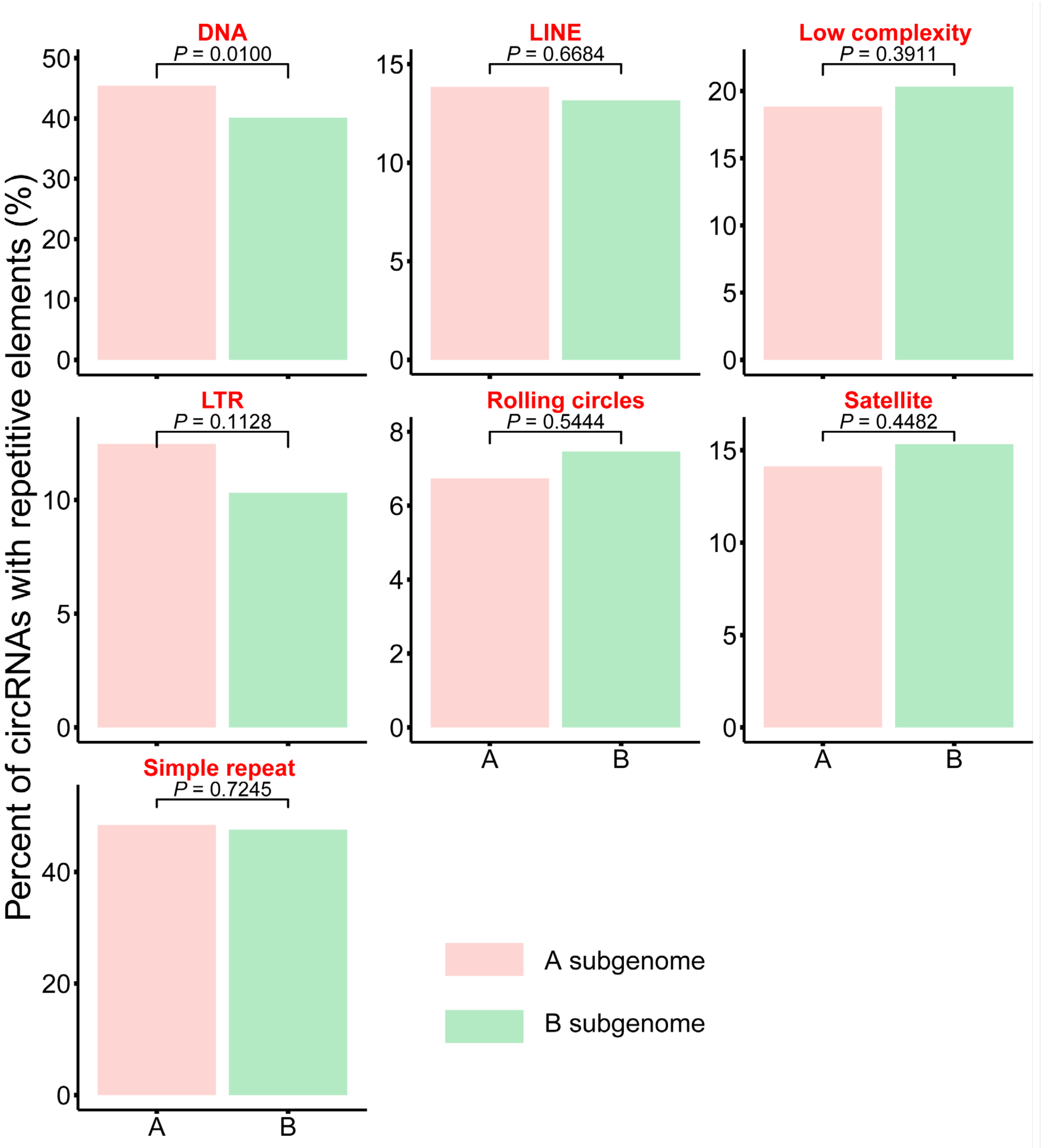

Comparison of percentage of flanking introns that contain transposon across the A and B subgenomes. *P* Values for chi-squared test was showed in figure.

Fig. S23. Expression profiling of circRNAs in nine organs

A

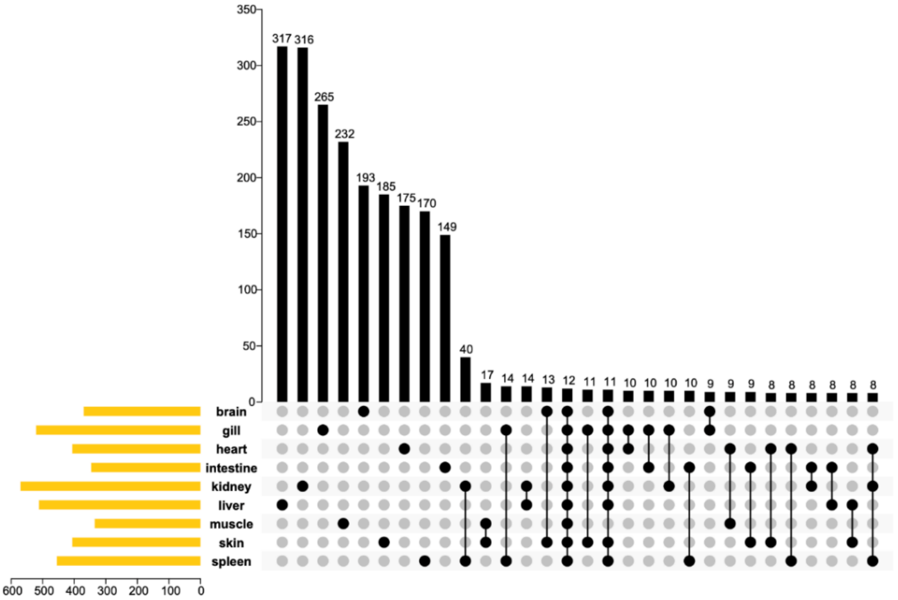

B

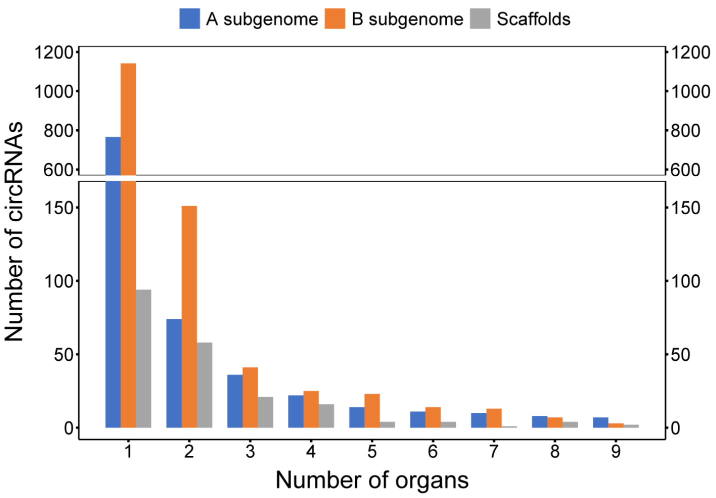

(A) Upset plot of circRNAs in nine organs. The orange bars on the left indicated the total number of circRNAs in each organ. The unique or shared circRNAs was represented by black dots connected by lines, and the top vertically aligned bar plot indicated the intersection size of circRNAs in nine organs. (B) Number of circRNAs shared with different organs.

Fig. S24. Number and base substitution frequency of various types of RNA editing sites

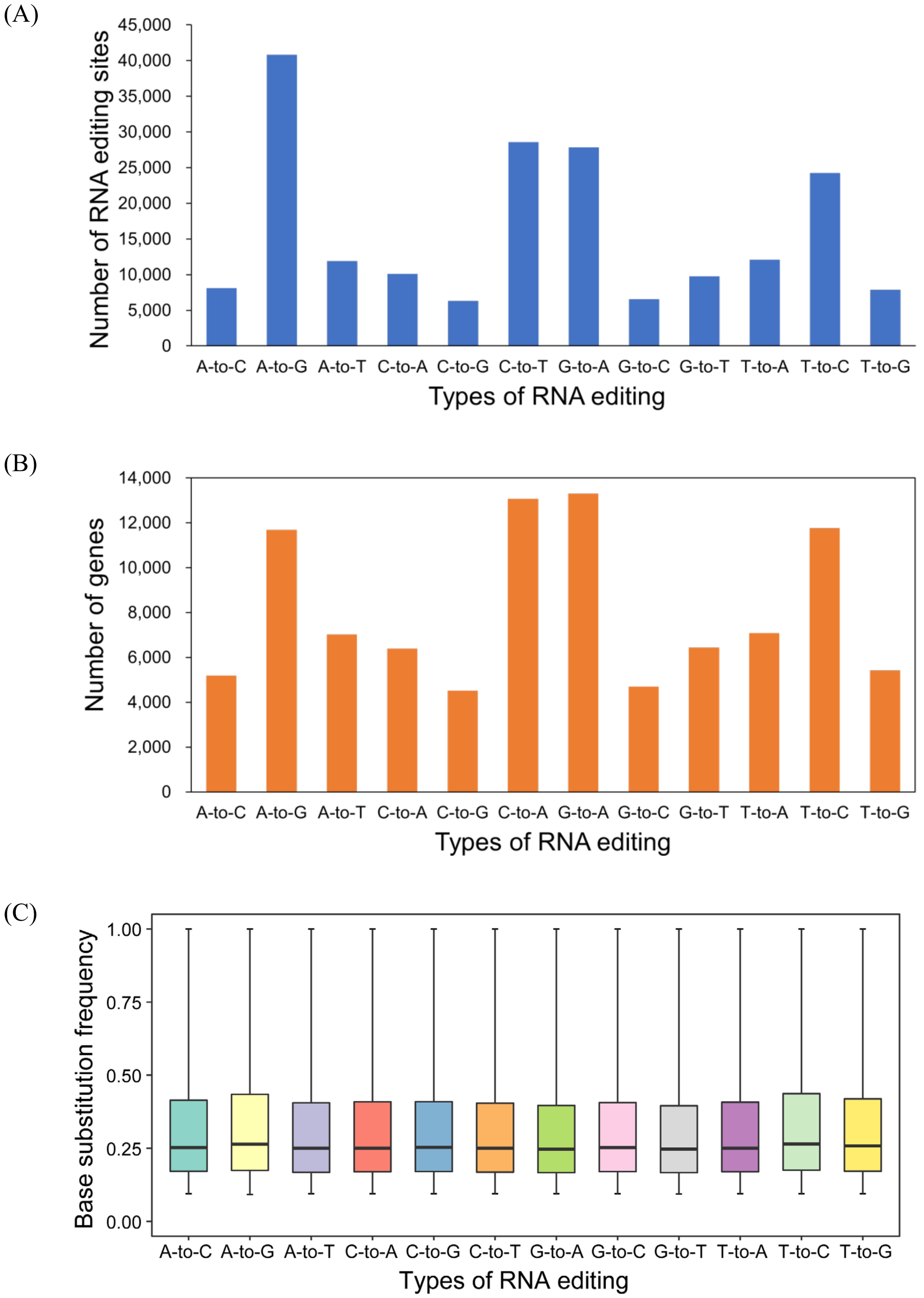

(A) The bar plot representing the number of different types of RNA editing. (B) The bar plot illustrating the number of genes undergoing different types of RNA editing. (C) The box plot depicting the base substitution frequency of different RNA editing types.

Fig. S25. Upset plot of RNA editing sites identified in nine organs

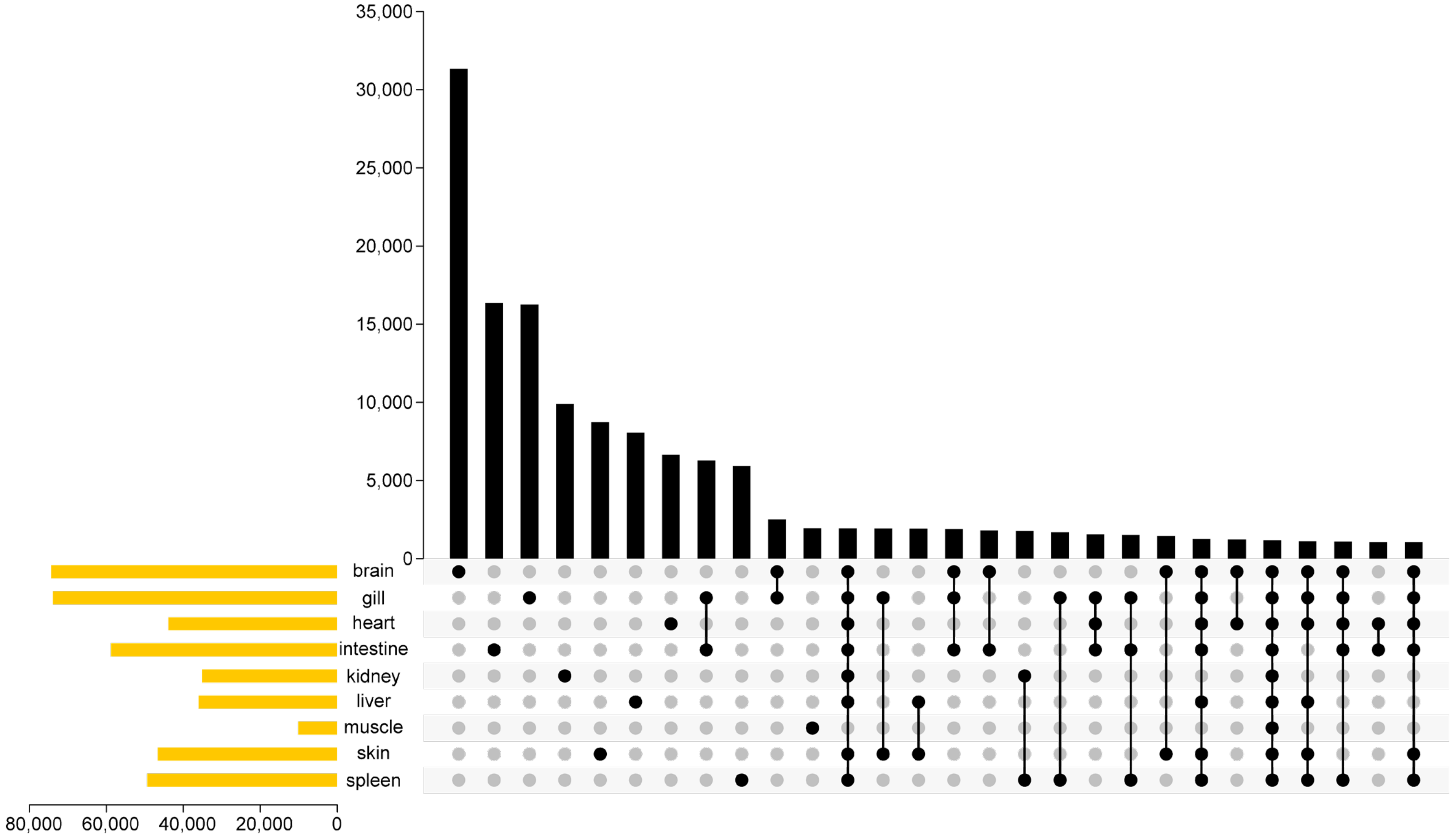

Upset plot showed the overlap of all RNA editing sites detected in nine organs. The orange bars on the left indicated the total number of RNA editing sites in each organ. The top vertically aligned bar plot indicated the intersection size of unique or shared RNA editing sites in nine organs, which were represented by black dots connected by lines.

Fig. S26. Statistics on the number of RNA editing sites in the A and B subgenomes of common carp

(A)

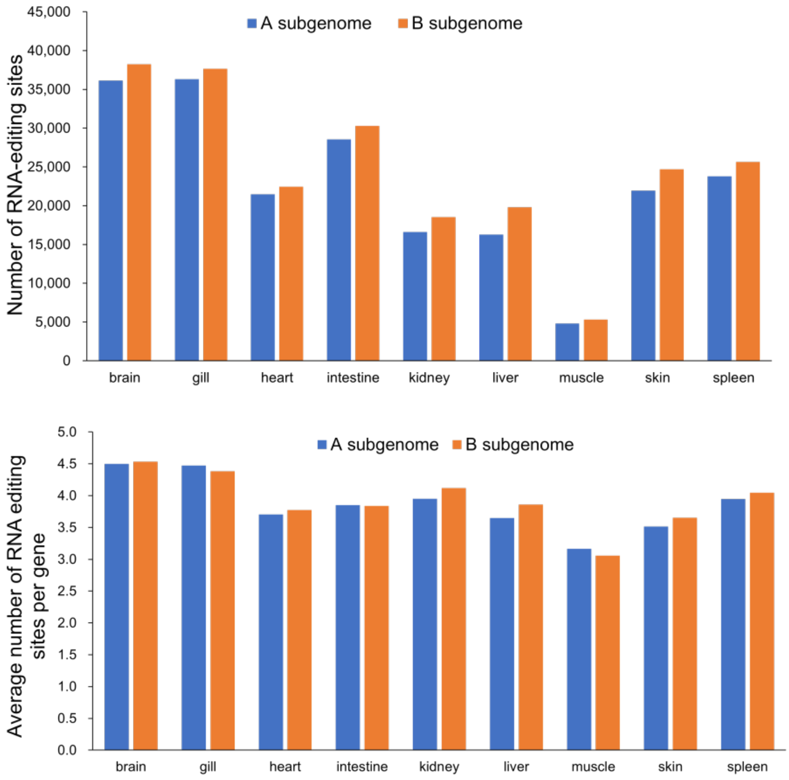

(B)

The bar plot showed the total number of RNA editing sites (A) and the average number of sites per gene (B) in nine organs of common carp.

Fig. S27. Base substitution frequency of RNA editing sites in homoeologous genes

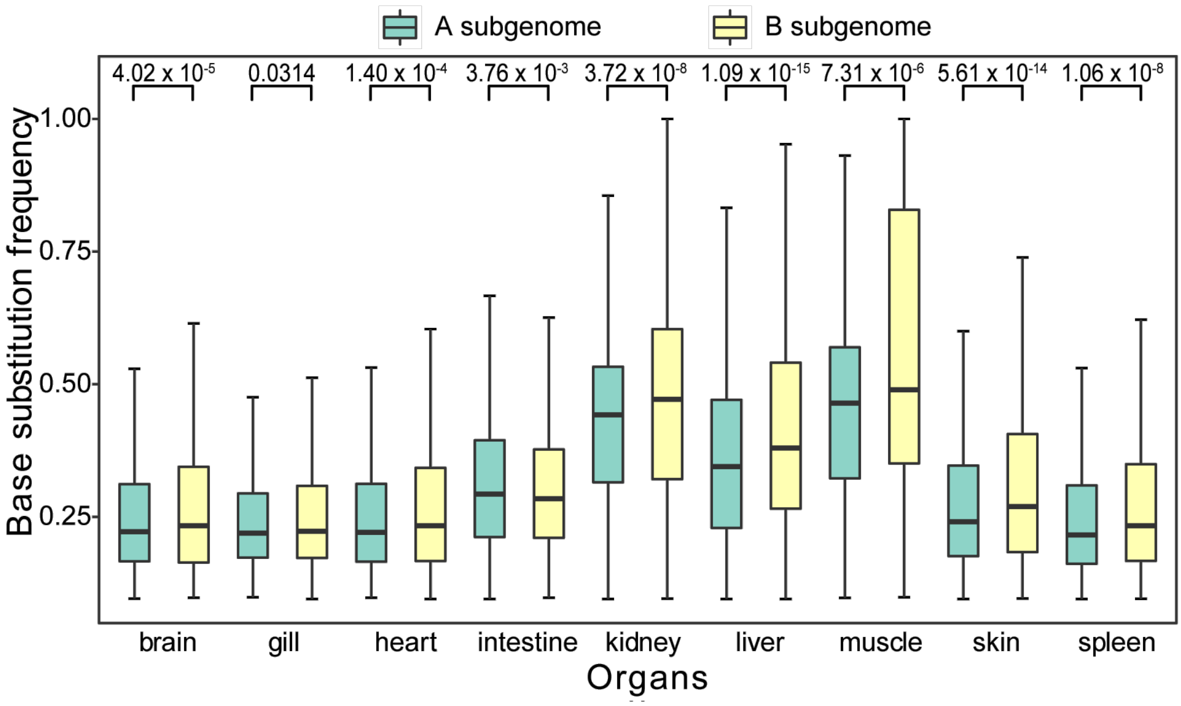

RNA editing sites on homoeologous genes in the B subgenomes showed higher base substitution frequency, as compared to the A subgenomes (Wilcoxon rank-sum test).

Fig. S28. Comparison of base substitution frequency of RNA editing sites shared by nine organs in common carp

A

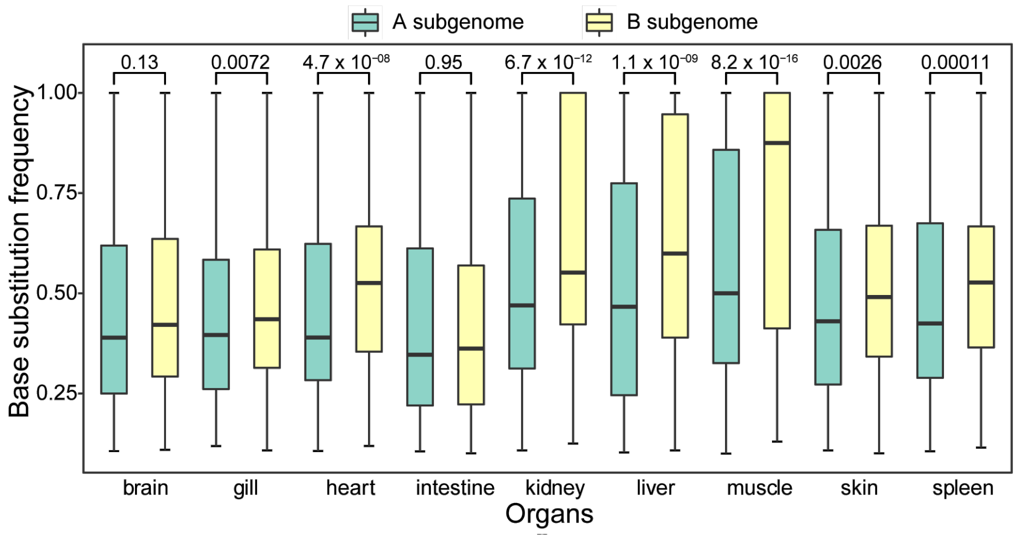

B

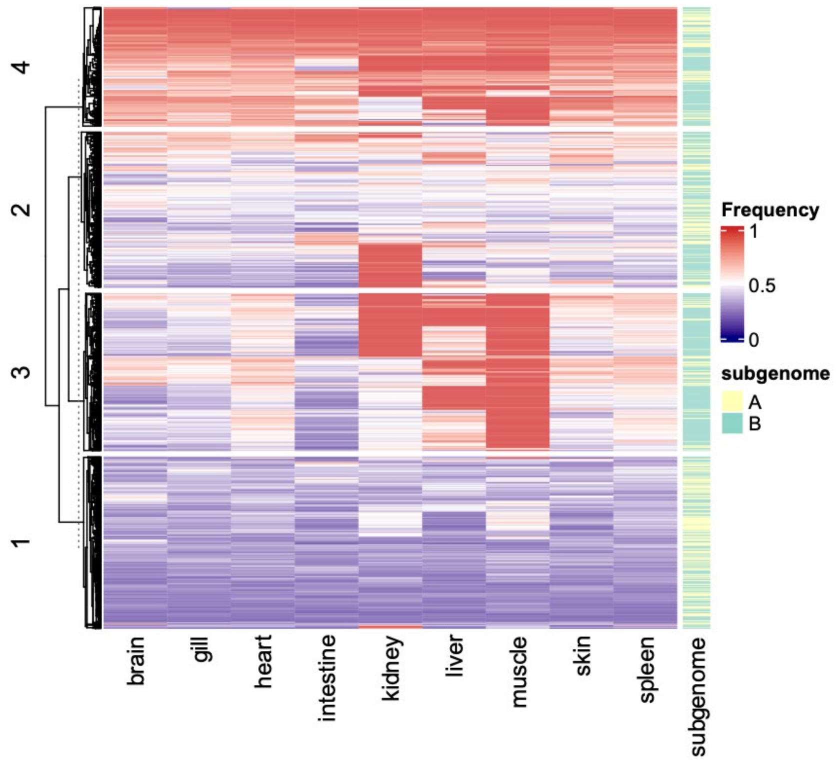

(A) The boxplot of base substitution frequency of the 1,173 RNA editing sites shared by nine organs (Wilcoxon rank-sum test). (B) The heatmap of RNA editing efficiency of the common sites.

Fig. S29. Enrichment of RNA editing sites in various genetic elements

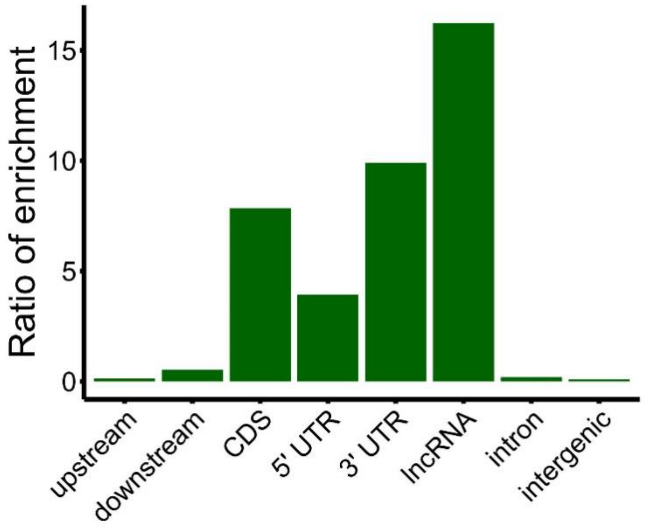

Enrichment of RNA editing sites in various genetic elements. The Y axis represents the enrichment ratio of RNA editing sites in different genetic elements. The enrichment ratio was calculated as ((The number of RNA editing sites from each genetic element category)/(Total number of RNA editing sites))/((Total length of each genetic element)/(Genome size)). RNA editing sites was preferred in lncRNA and 3’ UTR regions.

Fig. S30. Genome distribution of RNA editing sites in the A and B subgenomes

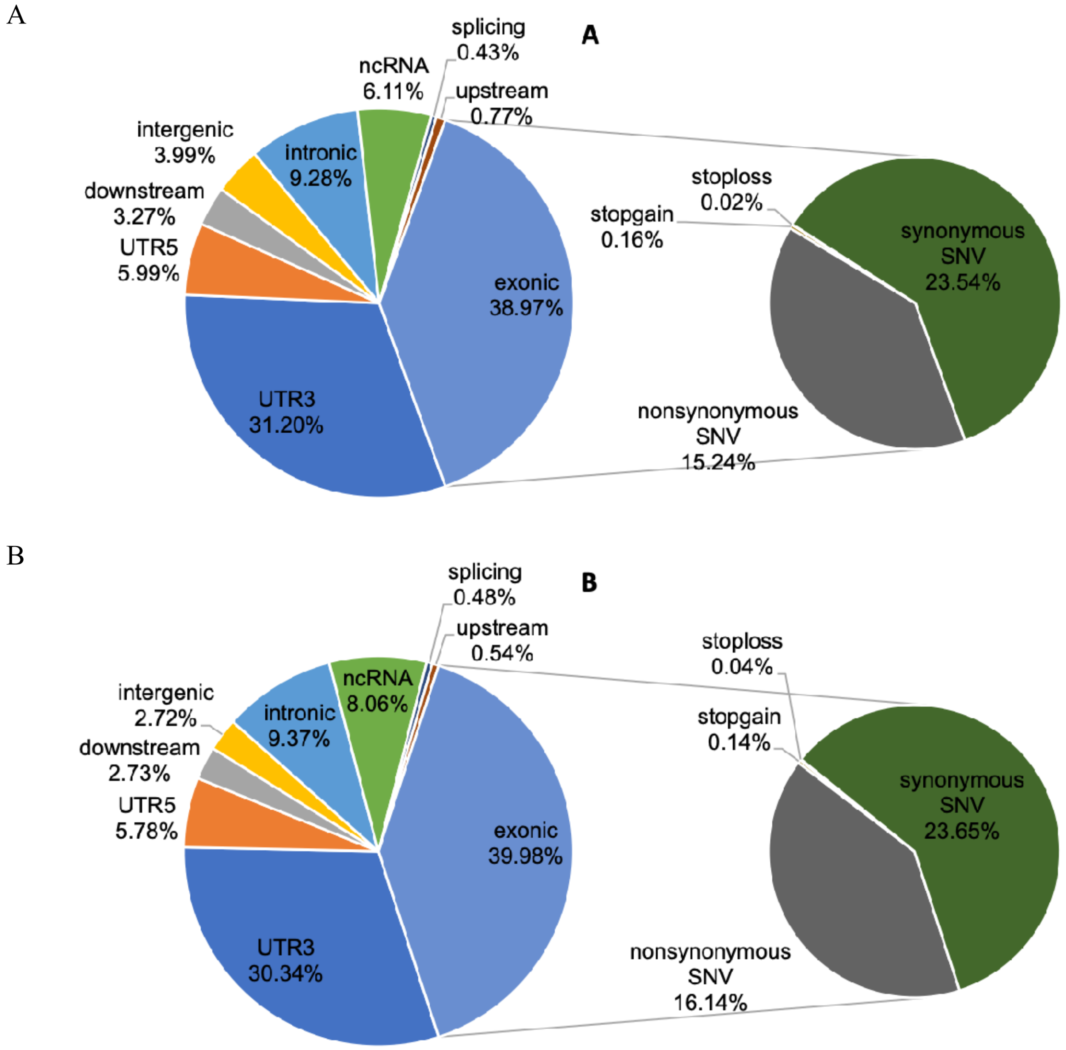

The figures showed the genome distribution of RNA editing sites in the A (A) and B (B) subgenomes, respectively.
