# Supplementary material for: An improved transcriptome annotation reveals asymmetric expression and distinct regulation patterns in allotetraploid common carp
Source: Commun Biol. 2024 Nov 20;7:1542. doi: 10.1038/s42003-024-07177-3 (PMC11579021; doi:10.1038/s42003-024-07177-3)
Supplement: Supplementary file 2 — Description of Additional Supplementary File [file 42003_2024_7177_MOESM2_ESM.pdf]

Description Of Additional Supplementary File

File name: Supplementary data

Description: Supplementary Tables 1-13
