# Supplementary material for: An improved transcriptome annotation reveals asymmetric expression and distinct regulation patterns in allotetraploid common carp
Source: Commun Biol. 2024 Nov 20;7:1542. doi: 10.1038/s42003-024-07177-3 (PMC11579021; doi:10.1038/s42003-024-07177-3)
Supplement: Supplementary file 4 — Reporting Summary [file 42003_2024_7177_MOESM4_ESM.pdf]

nature portfolio

Corresponding author(s): Jiong-Tang Li

Last updated by author(s): Oct 27, 2024

Reporting Summary

Nature Portfolio wishes to improve the reproducibility of the work that we publish. This form provides structure for consistency and transparency in reporting. For further information on Nature Portfolio policies, see our Editorial Policies and the Editorial Policy Checklist.

Statistics

For all statistical analyses, confirm that the following items are present in the figure legend, table legend, main text, or Methods section.

n/a	Confirmed	
☒	☐	The exact sample size (*n*) for each experimental group/condition, given as a discrete number and unit of measurement
☐	☒	A statement on whether measurements were taken from distinct samples or whether the same sample was measured repeatedly
☐	☒	The statistical test(s) used AND whether they are one- or two-sided<br>*Only common tests should be described solely by name; describe more complex techniques in the Methods section.*
☐	☒	A description of all covariates tested
☐	☒	A description of any assumptions or corrections, such as tests of normality and adjustment for multiple comparisons
☐	☒	A full description of the statistical parameters including central tendency (e.g. means) or other basic estimates (e.g. regression coefficient) AND variation (e.g. standard deviation) or associated estimates of uncertainty (e.g. confidence intervals)
☐	☒	For null hypothesis testing, the test statistic (e.g. *F*, *t*, *r*) with confidence intervals, effect sizes, degrees of freedom and *P* value noted<br>*Give P values as exact values whenever suitable.*
☒	☐	For Bayesian analysis, information on the choice of priors and Markov chain Monte Carlo settings
☒	☐	For hierarchical and complex designs, identification of the appropriate level for tests and full reporting of outcomes
☐	☒	Estimates of effect sizes (e.g. Cohen's *d*, Pearson's *r*), indicating how they were calculated

Our web collection on statistics for biologists contains articles on many of the points above.

Software and code

Policy information about availability of computer code

Data collection	No software was used for data collection.
Data analysis	GMAP v2020-06-30 was used for CCS reads alignment, and HISAT2 v2.2.1 was used to align RNA-seq reads to reference genome. Stringtie v2.2.1 was used to quantify the expression levels. TransDecoder v5.5.0 was used to predict protein sequences and KOBAS (online server) was used to annotated the KEGG pathways. Gene completeness was assessed using BUSCO v5.1.3. FEELnc was used to identify lncRNAs and CIRCexplorer2 v2.2.7 was used to identify circRNAs. REDItools v1.3 was used to detect RNA editing sites. TBtools v1.095 and R v4.1.1 was used to perform statistical analyses and visualization.

For manuscripts utilizing custom algorithms or software that are central to the research but not yet described in published literature, software must be made available to editors and reviewers. We strongly encourage code deposition in a community repository (e.g. GitHub). See the Nature Portfolio guidelines for submitting code & software for further information.

Data

Policy information about availability of data

All manuscripts must include a data availability statement. This statement should provide the following information, where applicable:

- Accession codes, unique identifiers, or web links for publicly available datasets
- A description of any restrictions on data availability
- For clinical datasets or third party data, please ensure that the statement adheres to our policy

The PacBio Iso-seq data were deposited in the NCBI SRA database under accession number PRJNA752470. The RNA-seq data of nine common carp organs were downloaded from the NCBI SRA database under accession number PRJNA684670. The improved transcriptome annotations and data supporting the results reported in the manuscript are available at Figshare (doi:10.6084/m9.figshare.25283650).

Research involving human participants, their data, or biological material

Policy information about studies with human participants or human data. See also policy information about sex, gender (identity/presentation), and sexual orientation and race, ethnicity and racism.

Reporting on sex and gender	We did not include any human research participants. To avoid sex-related differences, mixed samples were used for sequencing.
Reporting on race, ethnicity, or other socially relevant groupings	We did not include any human research participants. This item is not applicable to our work.
Population characteristics	We did not include any human research participants. This item is not applicable to our work.
Recruitment	We did not include any human research participants. This item is not applicable to our work.
Ethics oversight	We did not include any human research participants. This study was approved by the Animal Care and Use Committee of the Chinese Academy of Fishery Sciences (ACUC-CAFS, Permit Number: ACUC-CAFS-20180034).

Note that full information on the approval of the study protocol must also be provided in the manuscript.

Field-specific reporting

Please select the one below that is the best fit for your research. If you are not sure, read the appropriate sections before making your selection.

☒ Life sciences ☐ Behavioural & social sciences ☐ Ecological, evolutionary & environmental sciences

For a reference copy of the document with all sections, see nature.com/documents/nr-reporting-summary-flat.pdf

Life sciences study design

All studies must disclose on these points even when the disclosure is negative.

Sample size	For PacBio sequencing, six samples were collected and mixed.
Data exclusions	No data was excluded.
Replication	A mixture of six biological replicates was used to generate the sequencing data.
Randomization	Fish for RNA sequencing were sampled randomly.
Blinding	Blinding was not relevant for this study.

Reporting for specific materials, systems and methods

We require information from authors about some types of materials, experimental systems and methods used in many studies. Here, indicate whether each material, system or method listed is relevant to your study. If you are not sure if a list item applies to your research, read the appropriate section before selecting a response.

Materials & experimental systems

n/a	Involved in the study
☒	☐ Antibodies
☒	☐ Eukaryotic cell lines
☒	☐ Palaeontology and archaeology
☐	☒ Animals and other organisms
☒	☐ Clinical data
☒	☐ Dual use research of concern
☒	☐ Plants

Methods

n/a	Involved in the study
☒	☐ ChIP-seq
☒	☐ Flow cytometry
☒	☐ MRI-based neuroimaging

Animals and other research organisms

Policy information about studies involving animals; ARRIVE guidelines recommended for reporting animal research, and Sex and Gender in Research

Laboratory animals	We used common carp cultivated at the hatchery station of Chinese Academy of Fishery Sciences (Beijing, China)
Wild animals	We did not use any wild animals.
Reporting on sex	There were both male and female in our study, but all samples were mixed.
Field-collected samples	We did not use samples collected from the field.
Ethics oversight	The study was approved by the Animal Care and Use Committee of the Chinese Academy of Fishery Sciences (ACUC-CAFS, Permit Number: ACUC-CAFS-20180034), and conducted following the recommendations of the Care and Use of Animals for Scientific Purposes established by ACUC-CAFS.

Note that full information on the approval of the study protocol must also be provided in the manuscript.

Plants

Seed stocks	Not applicable.
Novel plant genotypes	Not applicable.
Authentication	Not applicable.
